# Nurturing the marriages of urinary liquid biopsies and nano‐diagnostics for precision urinalysis of prostate cancer

**DOI:** 10.1002/SMMD.20220020

**Published:** 2023-02-14

**Authors:** Caizhi Liao, Zhihao Wu, Chan Lin, Xiaofeng Chen, Yaqun Zou, Wan Zhao, Xin Li, Guangqi Huang, Baisheng Xu, Giovanni E. Briganti, Yan Qi, Xianshu Wang, Tao Zeng, Alain Wuethrich, Hongzhi Zou

**Affiliations:** ^1^ Creative Biosciences (Guangzhou) Co., Ltd Guangzhou China; ^2^ School of Environmental and Geographical Sciences Shanghai Normal University Shanghai China; ^3^ School of Chemistry Northwestern University Chicago Illinois USA; ^4^ Department of Urology Sir Run Run Shaw Hospital Zhejiang University Hangzhou China; ^5^ Peking University Health Science Center Beijing China; ^6^ Department of Urology The First People's Hospital of Xiushui Jiujiang China; ^7^ Department of Urology University of Florence Florence Italy; ^8^ Department of Urology the Second Affiliated Hospital of Nanchang University Nanchang China; ^9^ Centre for Personalised Nanomedicine, Australian Institute for Bioengineering and Nanotechnology, The University of Queensland Brisbane Queensland Australia; ^10^ The Sixth Affiliated Hospital Sun Yat‐sen University Guangzhou China

**Keywords:** nano diagnostics, nanomaterial, prostate cancer (PCa), urinary biomarker, urinary liquid biopsy

## Abstract

Prostate cancer remains the second‐most common cancer diagnosed in men, despite the increasingly widespread use of serum prostate‐specific antigen (PSA) screening. The controversial clinical implications and cost benefits of PSA screening have been highlighted due to its poor specificity, resulting in a high rate of overdiagnosis and underdiagnosis. Thus, the development of novel biomarkers for prostate cancer detection remains an intriguing challenge. Urine is emerging as a source for prostate cancer biomarker discovery. Currently, new urine biomarkers already outperform serum PSA in clinical diagnosis. Meanwhile, the advances in nanotechnology have provided a suite of diagnostic tools to study prostate cancer in more detail, sparking a new era of biomarker discoveries. In this review, we envision that future prostate cancer diagnosis will probably integrate multiplex nano‐diagnostic approaches to detect novel urinary biomarkers. However, challenges remain in differentiating indolent from aggressive cancers to better inform treatment decisions, and clinical translation still needs to be overcome.

## Graphical abstract

For prostate cancer diagnosis, challenges remain in differentiating indolent from aggressive cancers to better inform treatment decisions, and clinical translation still needs to be overcome. The future prostate cancer diagnosis will probably integrate multiplex nano diagnostic approaches to detect novel urinary biomarkers.

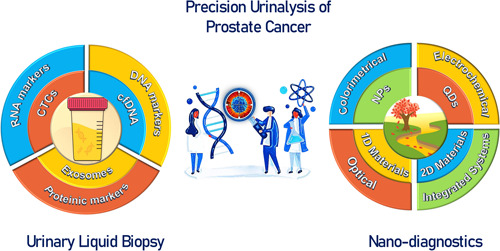

1

**Key points**

- Prostate cancer remains the second‐most common cancer diagnosed in men, despite the increasingly widespread use of serum prostate‐specific antigen (PSA) screening.
- The controversial clinical implications and cost benefits of PSA screening have been highlighted due to its poor specificity, resulting in a high rate of overdiagnosis and underdiagnosis.
- Urine is emerging as a source for the development of novel biomarkers for prostate cancer detection.
- The advances in nanotechnology have provided a suite of diagnostic tools to study prostate cancer in more detail, sparking a new era of biomarker discoveries.
- Future prostate cancer diagnosis will probably integrate multiplex nano‐diagnostic approaches to detect novel urinary biomarkers.

## 2 INTRODUCTION

Prostate cancer (PCa) represents the most frequent solid cancer in males, and it continues to be the primary cause of cancer‐related death in these groups.^1^, ^2^ Comprehensive multi‐omics investigations, including genomic, transcriptomic, epigenomic, and proteomic analyses, have been undertaken in the last few years to better understand the heterogeneity of this malignancy.^3^, ^4^, ^5^, ^6^ According to studies, a large percentage of castration‐resistant prostate cancers (CRPC) is still hormone‐driven: 40%–70% of prostate cancers contain chromosomal rearrangements that lead to the hormonal regulation of carcinogenic gene expression.^7^, ^8^

Prostate cancer cells rely heavily on androgen stimulation for their proliferation and survival. Men with incurable advanced prostate cancer frequently develop “metastatic” or “hormone‐refractory” CRPC, which is mostly determined by receptor transcriptional activity (AR).^9^, ^10^ Several oncogenic drivers have been linked to the development of prostate cancer, including I: Gene aberrations of cell‐signaling and genetic‐repairing genes (*e.g.*, PI3K, BRCA, and PTEN, *etc.*); II: Androgen receptor gene amplification and overexpression in hormone‐refractory prostate cancer, as well as copy number variations of androgen receptor in CRPC; III: Relevant gene point mutations (*e.g.*, F877L, TP53, and FOXA1, *etc.*); IV: Prostate cancer metastatic progression and germline variations. Alone with such discoveries as a refined understanding of prostate cancer, we now better comprehend the potential advantages of precision management of prostate cancers.^11^, ^12^, ^13^, ^14^, ^15^

Besides imaging techniques, commonly including magnetic resonance imaging (MRI), positron emission tomography (PET), and computed tomography (CT), a digital rectal exam (DRE) and prostate‐specific antigen (PSA) test have been widely included in prostate screening. If prostate cancer screening reveals an abnormality, other diagnostic methods, including a prostate biopsy, are used to confirm the findings.^16^, ^17^, ^18^ Prostate cancer‐related biomarkers are typically used to decide whether to perform a prostate biopsy, develop the best treatment regimens, and evaluate patient reactions during treatment.^5^, ^19^, ^20^ One of the most difficult aspects of precision prostate cancer screening is distinguishing between patients with clinically important subtypes that require aggressive treatment and those with indolent cancer types that are unlikely to grow and cause patient mortality.^7^, ^9^, ^19^, ^21^, ^22^ As such, mismanagement of prostate cancer screenings is a problematic issue that can lead to overdiagnosis and unnecessary or even fatal therapy.

Testing healthy males with no indications of prostate cancer remains contentious in light of this context. Whether the benefits of screening and testing outweigh the risks is a point of active debate amongst medical institutions.^23^, ^24^, ^25^ Current approaches to obtaining accurate detection and subsequent precision therapy of high‐grade aggressive prostate cancer types are still debatable. The blood‐based biomarker‐driven approach that measures serum prostate‐specific antigen (PSA) levels—also referred to as Gamma‐Semin protein or kallikrein‐3 (KLK3)—quickly developed into comprehensive screening programs after being proposed by Dr. Ta Stamey in the early 1980s and approved by the Food and Drug Administration (FDA) in 1986.^17^, ^26^, ^27^, ^28^ PSA levels in the blood can signal prostate cancer, and their usage has the potential to change prostate cancer management by allowing for early detection and therapy intervention, potentially reducing malignant death. PSA population screening reduced high‐ and intermediate‐risk patients from 68.9% to 52.3%, resulting in a 21% reduction in prostate cancer mortality, according to the study.^16^, ^29^, ^30^

However, mounting evidence casts doubt on the utility of PSA as a prostate cancer screening biomarker: Other factors, including enlarged prostate and certain drugs, could also alter the PSA levels. PSA screening is therefore associated with a high likelihood of false‐positive diagnoses (70%) or false‐negative diagnoses (20%), as well as ambiguous or even misleading screening results, all of which can lead to over/under‐diagnosis and unnecessary treatment.^7^, ^17^, ^28^, ^31^, ^32^, ^33^ It has become evident that neither PSA nor DRE, alone or in combination, are adequate for assessing and targeting clinically relevant prostate cancer. As a result, there is a requirement for enhanced prostate cancer screenings and improved detection of aggressive malignancy subtypes.^25^, ^34^, ^35^, ^36^ Despite the lessons learned from the huge PSA screens, developing viable alternative biomarkers for precision prostate cancer diagnosis continues to be a difficult task.

Urine is a complex liquid waste that contains a variety of substances, some of which are filtered from circulation, such as metabolic waste products and small proteins secreted by different cell types, as well as larger proteins and cells originating from urogenital organs downstream of glomerular filtration.^37^, ^38^, ^39^ Urine has shown promise as a reliable noninvasive source of cancer biomarkers.^40^ Urine has several advantages over other clinical biological specimens (*e.g.,* plasma or serum) for the identification of diagnostic and prognostic biomarkers: I) Easy to collect in large volumes, noninvasive, and safe for direct contact of human body; II) no major proteolytic degradation; and III) less complicated composition than plasma/serum, minimizing interferences in urine isolation and facilitating the discovery of novel biomarkers.^40^, ^41^, ^42^, ^43^

Particularly, great efforts have been devoted to identifying potential prostate cancer biomarkers in the urine.^39^, ^42^, ^44^, ^45^, ^46^ Urinary markers have received a lot of attention in recent prostate cancer screenings as a supplement to serum PSA levels to avoid tissue biopsies, as a complementary tool to biopsy analysis, or even as a gold standard method that completely substitutes the biopsy procedure.^47^, ^48^, ^49^ A slew of recent research has suggested that reliable urine prognostic and predictive markers have the potential to assist clinical decision‐making by distinguishing between benign and malignant prostate diseases.^21^, ^23^, ^40^, ^50^, ^51^

An ideal urine test would aid in the diagnosis of clinically significant early‐stage prostate tumors while minimizing the detection of benign subtypes, allowing for correct classification and stratified therapy of prostate cancer. Various markers, including genetic‐based, exosome‐based, and circulating tumor cells‐based markers, have shown promise in identifying and predicting the aggressiveness of prostate cancer over the last decade, according to a growing body of biomarker discovery programs employing urine samples.^4^, ^7^, ^46^, ^52^, ^53^, ^54^, ^55^, ^56^, ^57^ These urine prostate cancer markers have advanced through the discovery and development stages and, more significantly, have been shown to be clinically useful in multicenter investigations.

For the measurement of urine biomarkers, laboratory polymerase chain reaction (PCR)‐based approaches are commonly used. PCR‐based methods have several drawbacks, including time‐consuming operation, easy contamination, and high costs, all of which have been identified as impediments to the next stage of development of urinary analysis, the corner stone of the next‐generation of precision prostate cancer management. Complementary easy‐to‐use and low‐cost analytical approaches are in high demand, given the potential translational utility of urine prostate cancer biomarkers.

Nanotechnology is a new field that entails the development and manipulation of materials at the nanoscale level to create products with unique qualities.^58^, ^59^ Nanotechnology is predicted to alleviate the current restrictions of molecular analysis in the laboratory by delivering speedy, cost‐effective, and sample‐to‐answer clinical diagnostic tools.^60^, ^61^ Nano‐diagnostics, or the marriage of nanotechnology and cancer prognosis/diagnosis, encourages continuous advancements in clinical diagnostics, ranging from emerging nanopore sequencers with real‐time long‐reading capabilities to integrated miniaturized systems for precision assessment of next‐generation biomarkers. Promisingly, many of these nanotechnologies have already undergone extensive clinical testing for cancer detection applications.^25^, ^58^, ^62^, ^63^, ^64^, ^65^ The introduction of nano‐diagnostic techniques has the potential to unlock the door to possible cancer screenings, according to promising results.

**FIGURE 1:**
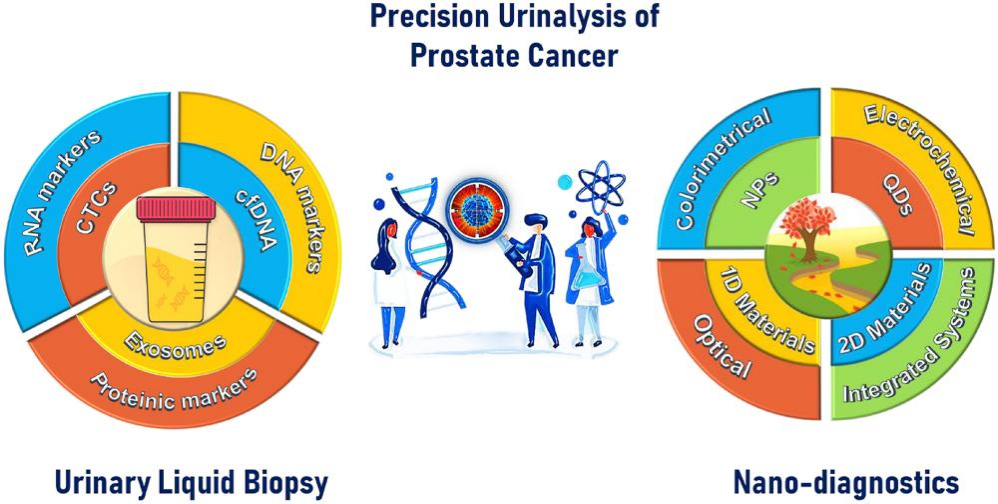
Schematic presentation of precision prostate cancer management enabled with the combination of urinary liquid biopsy and nano‐diagnostics. Circulating tumor cells (CTCs), cell‐free DNA (cfDNA), and exosomes, severing as the troika of urinary liquid biopsy, carry three key types of urinary biomarkers, including RNA‐subtype markers, DNA‐subtype markers, and protein‐subtype markers (left figure). Promising nanomaterials, exemplified with nanoparticles (NPs), quantum dots (QDs), one‐dimensional materials (1‐DMs), and two‐dimensional materials (2‐DMs), establish the cornerstone for the novel nano‐diagnostic platforms, including colorimetrical, electrochemical, optical, and integrated all‐in‐one systems. The integration of urinary liquid biopsy and nano‐diagnostics shepherds the idea of precision cancer management from concept to launch.

This review attempts to envision a tale of two intriguing players: urinary liquid biopsy and nanotechnology‐based diagnostic paradigms, see Figure 1. To inspire next‐stage research in this exciting field, we strive to feature the latest advancements in the development of noninvasive urinary biomarkers, including genetic markers, and proteomic markers, and critically discuss the current evidence concerning the applicability of these candidates as diagnostic, prognostic, and predictive biomarkers in prostate cancer (Section 2). In Section 3, we will systematically discuss the nanotechnology‐based approaches, including colorimetric, electrochemical, optical, and integrated sample‐to‐answer systems. Merging nano‐diagnostic methods with potential urinary biomarkers could remarkably boost the precision risk management of prostate cancer. In the last part (Section 4), we will crystalize key findings, pinpoint challenges that remain to be addressed, and present an outlook on the future synchronized development of nano‐diagnostics and urinary biomarkers in prostate cancer detection. Promisingly, this review bridges the knowledge gaps between nano‐diagnostic and prostate cancer clinical research fields, hopefully adding to the armamentarium of prognostic, diagnostic, and predictive tools for prostate cancer.

## 3 URINARY LIQUID BIOPSY

Biopsies have been used to diagnose and manage the disease for 1000 years.^66^, ^67^, ^68^ As the techniques that have enabled us to analyze a biopsy become ever more sophisticated, we have realized the limitations of looking at this single snapshot of the tumor. A traditional solid biopsy is invasive and is challenging to obtain in certain tumors. Furthermore, a single solid biopsy could not provide a comprehensive view of the tumor's genetic and morphologic landscapes, as many tumors are genetically and morphologically heterogeneous with numerous subclones. Considering these limitations on the use of tissue biopsies, new approaches to studying tumor genetics and tumor dynamics have emerged. Among these, liquid biopsy represents a revolutionary technique that is revealing previously unimaginable possibilities.^37^, ^38^, ^69^, ^70^

**FIGURE 2:**
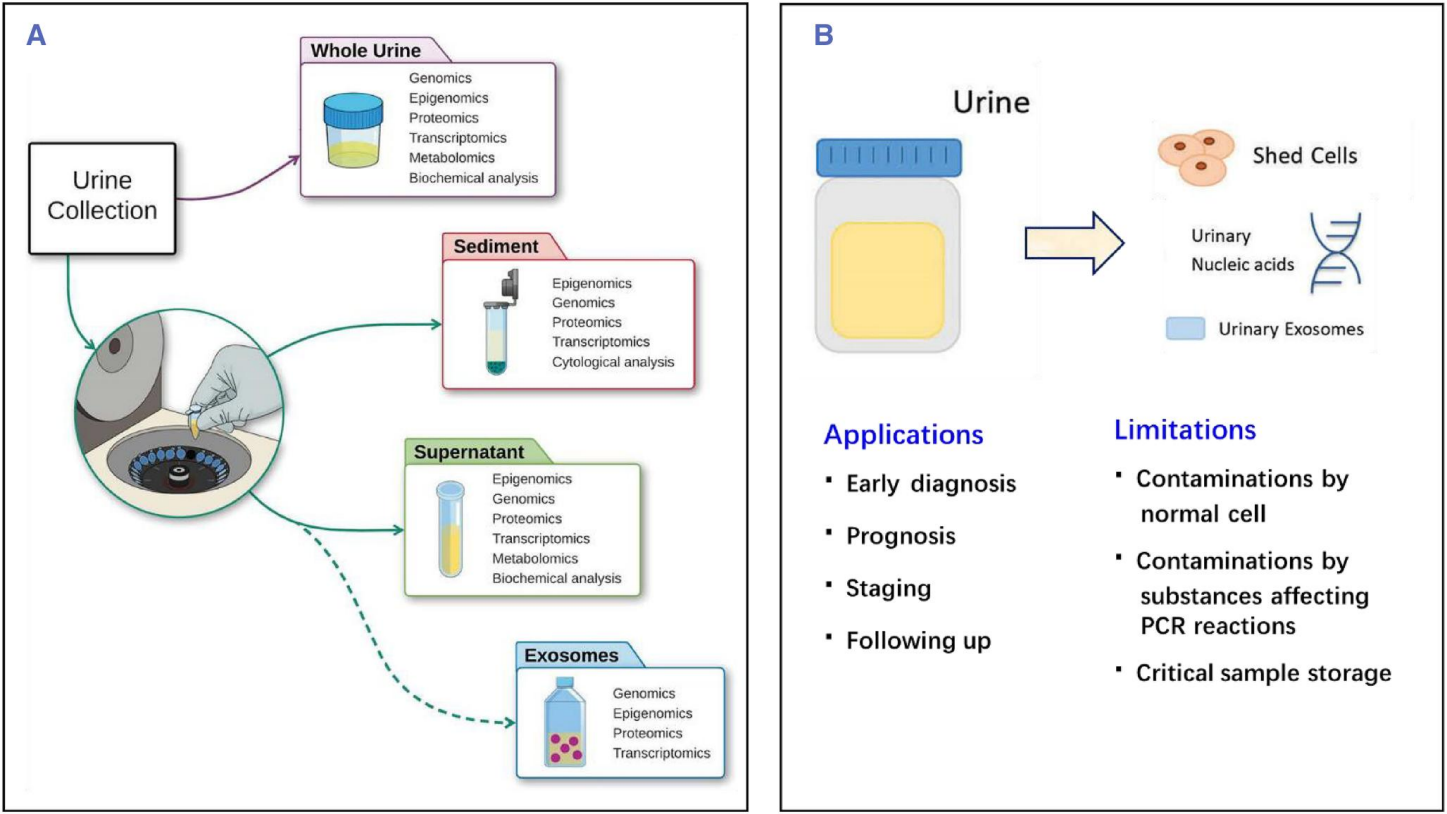
(A) Urine processed with centrifugation is divided into several distinct parts, including sediment, supernatant, and exosomes, all of which carry multi‐omics urinary biomarkers for prostate cancer analysis. Reproduced with permission.^73^ Copyright 2019, Springer Nature Limited. (B) Sources of urinary biomarkers, application, and limitations in urine liquid biopsies. Reproduced with permission.^69^ Copyright 2020, Taylor & Francis Group.

As an emerging noninvasive form of liquid biopsy without introducing any risk of physical harm to the patient, urinary liquid biopsy has garnered particular attention in the search for promising biomarkers of prostate cancer.^71^ Urine contains assorted cancer‐associated elements that are found to be released directly from the prostate gland into urine including cell‐associated markers and secreted cell‐free markers, projecting a rosy future with precision management of prostate cancer being attained,^40^, ^72^ see Figure 2.

### 3.1 The troika of urinary liquid biopsy

Urine samples can be separated into urinary sediment and urine supernatant via centrifugation.

**FIGURE 3:**
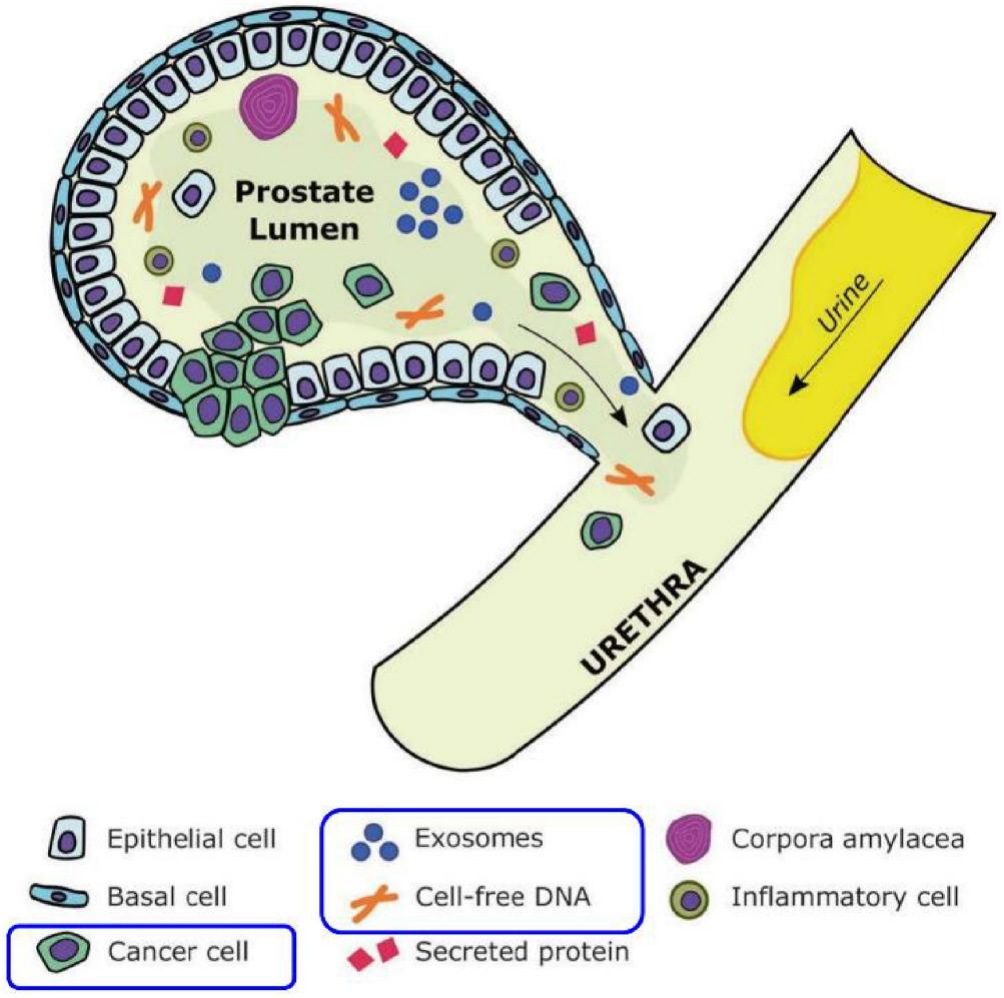
Schematic presentation of promising prostatic components detectable in urine, as exemplified by circulating tumor cells (CTCs), cell‐free DNA (cfDNA), and exosomes, all of which could carry different types of urinary biomarkers for PCa analysis. Reproduced with permission.^73^ Copyright 2019, Springer Nature Limited.

Urine samples, rather than blood samples, could be better suited for individuals with early localized prostate cancer since blood samples contain biomarkers from almost all body tissues, resulting in substantial background interference that reduces detection performance.^37^, ^74^, ^75^ It's worth noting that the analysis of urine liquid biopsy includes the separation and detection of three key components: circulating tumor cells (CTCs),^8^ circulating tumor DNA (ctDNA),^76^, ^77^ and exosomes or extracellular vesicles (EVs).^78^ These three dominating components, the troika of urine liquid biopsy, carry urinary biomarkers (*i.e.*, proteins, DNA, and RNA) and therefore unfold new possibilities for prostate cancer precision management as a source of genetic and proteomic information in patients with prostate cancer (Figure 3).

#### 3.1.1 Circulating tumor cells (CTCs)

A typical malignant tumor contains millions or even billions of cells harboring genetic mutations that drive cellular growth, aberrant division, and invasion of the surrounding tissues. However, as the cells proliferate and the tumor grows, some cells, referred to as CTCs, shed from the primary tumor to form potentially dangerous metastatic sites. CTCs are thought to be enriched in metastatic precursors. Once in the circulation, however, CTCs seem to persist for a short time only (half‐life: 1–2.4 h).^79^ CTCs are now obtained using more sophisticated technology for a variety of clinical purposes, including evaluating disease burden and detecting minimal residual disease, as well as profiling CTCs as a way to target therapy to these putative culprit cells.^80^, ^81^

CTCs were first discovered in the blood of a patient with extensive breast cancer in an autopsy in 1869, but they were not a common focus in cancer research until recently.^80^ CTC analysis has been particularly difficult because of their ultra‐rare nature, with levels as low as one CTC per 10^6^–10^7^ leukocytes in the peripheral blood of cancer patients.^82^, ^83^ The Cell Search System (Veridex, Raritan, NJ) was introduced in 2004, and it is now the only medical device approved by the Food and Drug Administration (FDA) for CTC selection and enumeration.

The scarcity of CTCs—which is estimated to be one CTC per 10^6^–10^7^ white blood cells (WBCs)—remains the biggest obstacle to CTC research. Sensitive methods that can effectively distinguish these cells from the millions of other blood cells are needed in order to identify and isolate rare CTCs from the bloodstream. Therefore, researchers are still dealing with a number of challenges, including methodological limits imposed by the Cell Search equipment, physics, and statistics,^35^, ^84^ as well as translational issues,^85^ which are limiting the clinical use of CTC assays. Even though several issues still exist that limit the current use of this burgeoning diagnostic approach, CTC analyses are ushering in a new age in oncology, where “real‐time” monitoring of tumor status is vital for effective therapy.^66^, ^81^

Distant metastasis during tumor development is the most common reason for therapy failure in most PCa patients.^86^ Metastasis requires cancer cells to circulate in the peripheral blood system in order to spread to distant organs with bone and blood metastasis prevailing.^87^, ^88^

Castration‐resistant PCa (CRPC) patients can have their androgen receptor (AR) and other associated genes measured at the subcellular level in CTCs from their peripheral blood. CTC enumeration has been studied extensively in both localized and metastatic prostate cancer. Initial hopes that identifying CTCs could predict disease recurrence in localized diseases were dashed. Davis and colleagues looked at individuals who had localized prostate cancer and had a radical prostatectomy. Only around 5% of the patients had more than two CTCs, and there was no link between CTC count and tumor volume, clinical stage, or Gleason score.^89^ CTCs were found in only one of 20 patients with high‐risk localized prostate cancer in another investigation using the Cell Search enrichment platform (no CTCs were found in healthy controls).^90^

In the case of metastatic PCa, where a higher number of CTCs is expected due to the larger volume of disseminated cancer, CTCs were found to provide better prognostic value. Before androgen deprivation therapy, 55% of men with metastatic prostate cancer had 5 CTCs/7.5 ml blood, according to Okegawa *et al.* In comparison to individuals with 5 CTCs who responded to ADT for 32 months (*P* = 0.007), these patients with less CTC count only responded to androgen deprivation (by PSA) for 17 months.^91^ CellSearch revealed a favorable association between CTC count, lactate dehydrogenase (LDH), and alkaline phosphatase, but not with PSA or testosterone levels, in a study of the same group. The median CTC count of patients who developed castrate resistance was 17 CTCs/7.5ml blood, while the median CTC count of those who did not develop castrate resistance was 1 CTC/7.5ml blood. Only baseline CTC numbers were indicative of progression to castration resistance in multivariate analysis (*P* < 0.001).^92^ A paradigm shift toward a more aggressive initial management of metastatic prostate cancer has occurred as a result of recent evidence supporting the early administration of chemotherapy with hormonal therapy.^93^

#### 3.1.2 Cell‐free DNA (cfDNA)

The development of noninvasive liquid biopsy technologies based on cell‐free DNA (cfDNA) analysis has opened the door to a new generation of diagnostic procedures. Although circulating cfDNA was discovered more than 50 years ago, anomalies in cancer patients were discovered only decades later, revealing that they contain greater levels of cfDNA.^94^, ^95^ A portion of cfDNA in cancer patients is tumor‐derived, and this is referred to as circulating tumor DNA (ctDNA). In theory, ctDNA analysis has the benefit of detecting tumor‐specific mutations. However, ctDNA analysis for solid tumors has been limited by the low levels of ctDNA in circulation. Promisingly, next‐generation sequencing (NGS) combined with advanced computational approaches has recently enabled ctDNA‐based tumor genotyping in a number of cancer types^96^, ^97^

Metastatic castration‐resistant prostate cancer (mCRPC) is a fatal disease that develops as a result of androgen deprivation therapy for castrate‐sensitive prostate cancer. The mutational landscape of mCRPC is complex, dynamic, and poorly defined, and following genomic alterations in patients with this disease can be difficult due to the requirement for repeated, intrusive biopsy samples. New research reveals that analysis of ctDNA, which is detectable in blood samples from virtually all patients with mCRPC, can yield clinically useful genetic information. Nonmalignant and cancer cells both shed cfDNA into the circulation, but the proportion of ctDNA in mCRPC patients is frequently greater than 1%, allowing for thorough tumor genome profiling.^98^, ^99^, ^100^ Several recent investigations have shown that mutations and copy number alterations in mCRPC patients' plasma ctDNA are compatible with somatic landscapes identified through metastatic tissue profiling.^9^, ^101^ Researchers investigated the therapeutic potential of ctDNA in 65 men with mCRPC who were given enzalutamide for 12 weeks and found that 38% of them reacted (as measured by changes in blood PSA levels) with a median progression‐free survival of 3.5 months. Before and after 12 weeks of enzalutamide treatment, blood samples were taken. ctDNA was found in 122 of the 125 samples taken from these patients, and 119 of these could be sequenced. Researchers employed microarray‐based comparative genomic hybridization to monitor mutations in 19 different prostate‐cancer‐associated genes, and PCR to detect frequent androgen receptor (AR) alterations, unveiling a handful of clinical information for the decision‐making in mCRPC management.^3^, ^70^, ^98^

Although ctDNA is currently the subject of intensive research and offers a promising “real time” tool for tumor characterization, there are still several obstacles to be addressed before it can be routinely used by clinicians. Sensitivity and specificity are the two primary issues for ctDNA analysis. Due to the ultra‐low level of ctDNA in blood, ctDNA is usually undetectable in peripheral blood. Furthermore, the results of ctDNA analysis may be false negative because the current PCR technique prefers to measure comparatively longer fragments, that is, >120 bp, while smaller fragments derived from tumors could not be identified effectively.^80^ Besides, both sample handling and liquid biopsy analysis lack a standard, established procedure, which could drastically impact the results.

#### 3.1.3 Extracellular vesicles

Small membrane‐bound vesicles released by most cells are known as extracellular vesicles (EVs). EVs have been recovered from a range of cell types, including immune cells,^102^ stem cells,^103^ neurological system cells,^104^ and various tumor cells.^105^ We use the term EVs in this article to refer to all types of vesicles found in the extracellular space, including exosomes and microvesicles, as recommended by the International Society for Extracellular Vesicles.^106^

EVs were first defined as vesicles released into the extracellular space by reticulocytes' multi‐vesicular bodies (MVBs). Initially, they were thought to be a process by which cells ejected unwanted chemicals into the extracellular space.^107^ EVs were discovered as early as the 1960s, but their significance remained obscure for a long time. EVs act as messengers by which cells communicate both locally and afar by transferring proteins, lipids, and nucleic acids such as DNA, mRNA, and microRNA (miRNA).^108^ In the context of tumor biology, mounting data suggests that EVs play a key role in tumor‐microenvironment communication; tumor cells can change the function of both local and distant normal cells by transferring EV cargo, increasing tumor development, and metastasis.^109^

Tumor‐derived EVs have emerged as prospective biomarkers having the potential to be used not just to monitor cancer progression but also as future therapeutic targets. Because cancer‐derived EVs may be detected in all body fluids, including blood, urine, mucus, and bronchial fluids,^110^ liquid biopsy can be used to test their potential as biomarkers. The noninvasive method of acquiring cancer derived EVs enables a longitudinal sampling of patients, which aids clinical decision‐making about early diagnosis, prognosis, cancer recurrence, medication resistance, and changes in the cancer oncogenic repertoire in possibly all malignancies. When EVs, particularly sEV protein and nucleic acid biomarkers, are used in clinical practice, a large number of studies have shown that they can be used as cancer biomarkers.^111^ While sEV lipids and other biomolecules have some clinical utility, the technical platforms now available do not allow for the successful translation of findings into a clinical setting.^112^, ^113^, ^114^

Wiggins *et al.* used electron microscope images to document the existence of EVs in urine for the first time in 1986.^115^ Urinary EVs have long been thought to come from cells in the urogenital tract. As a result, EVs are a potential source of molecular biomarkers for disorders of the urogenital tract, including prostate cancer. Nilsson *et al.* discovered two prostate cancer RNA biomarkers, PCA3 and TMPRSS2:ERG, in EVs extracted from the urine of PCa patients in 2009.^116^ Since then, a rising body of evidence suggests that urine EVs can be utilized to diagnose PCa, particularly high‐grade illness. The combination of normalized PCA3 and ERG RNA levels in urine EVs, as well as the standard of care (SOC), was enough to distinguish GS 7 PCa (AUC = 0.803).^117^ miRNAs have a different expression pattern in urine EVs recovered from patients with PCa compared to urine EVs isolated from healthy control males, according to next‐generation sequencing. miR21, miR204, and miR375, the three most differentially expressed miRNAs, were found and had an AUC of 0.866 for diagnostic performance in prostate cancer patients.^118^ A proteomic study of urine EVs using mass spectrometry identified proteins that were differentially expressed in PCa patients compared to healthy male controls. The use of multiple biomarkers including transmembrane protein 256 (TMEM256), late endosomal/lysosomal adaptor, MAPK, and MTOR activator 1 (LAMTOR1) improves PCa detection, providing an AUC of 0.94.^119^

EVs have attracted enormous attentions for cancer diagnosis; however, current EV‐based liquid biopsy is limited in practical clinical uses because of their size heterogeneities (30 nm to 1 mm) and various interference small extravesicular molecules (such as RNAs and proteins) in human biofluids.^120^ Effective EV isolation techniques and purification procedures are required before clinical analysis.

### 3.2 Urinary biomarkers

**FIGURE 4:**
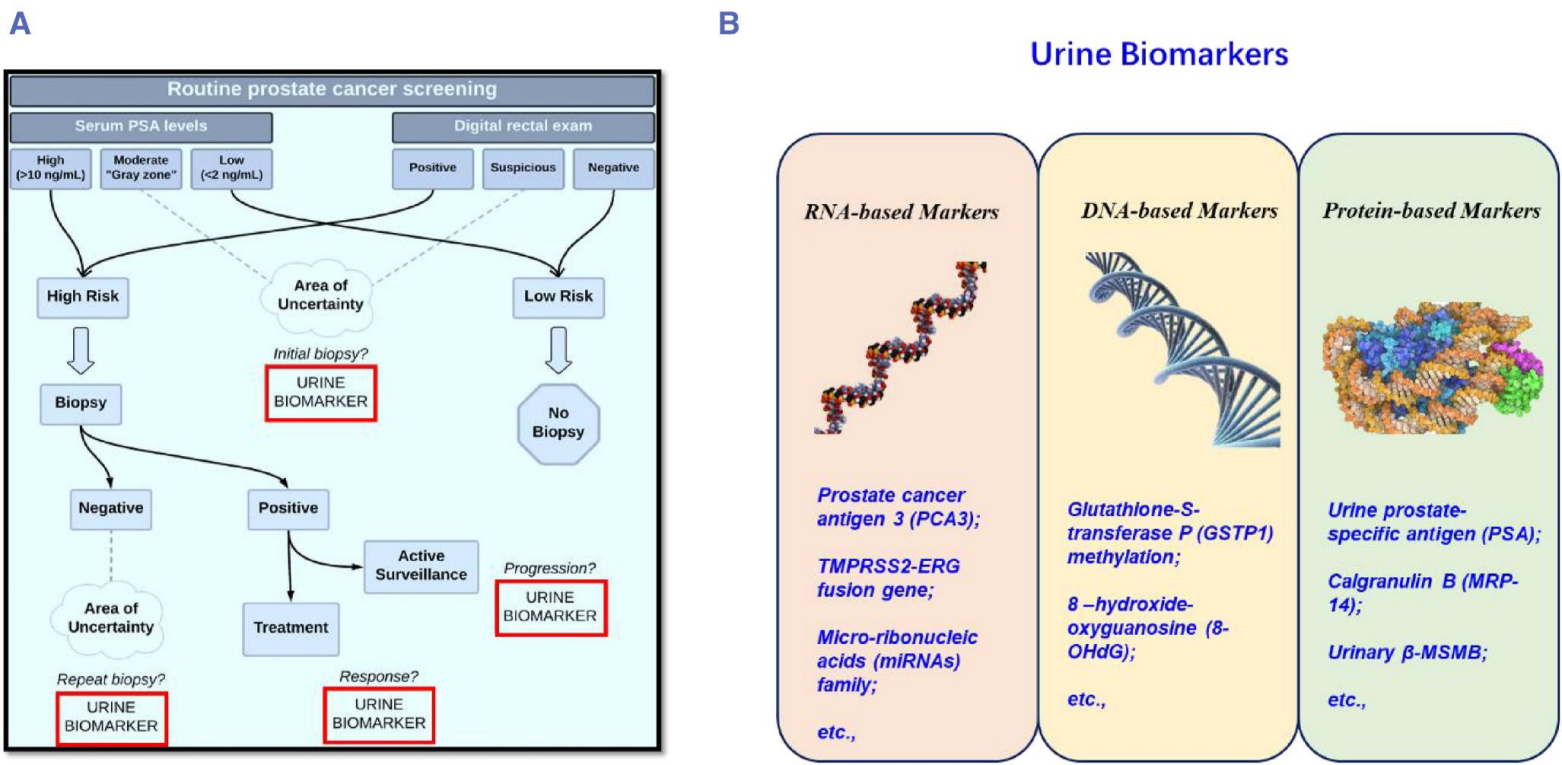
(A) The potential of urine biomarkers to improve PCa screening guides clinical decision‐making for precision management of PCa. Reproduced with permission.^73^ Copyright 2019, Springer Nature Limited. (B) Urinary biomarkers, including RNA markers, DNA markers, and protein markers, serve as candidates for improved PCa analysis.

Patients with aggressive tumors who can benefit from treatments can be distinguished from those with indolent tumors who should avoid overdiagnosis and inappropriate therapy using urine biomarkers linked to high‐risk traits. Ongoing research has a lot of potential for the future of molecular testing of urine biomarkers, which is one of the most promising clinical tools,^37^, ^38^, ^40^ see Figure 4. Extracellular‐derived nucleic acids that are secreted from cancer cells, such as RNA and DNA, were identified in the urine supernatant in high‐risk groups.^73^, ^74^ Because the transformation of a normal cell into a malignant cell is usually accompanied by profound genetic changes, epigenetically active long noncoding RNAs (Inc RNAs), such as PCA3, DNA epigenetic modifications, such as methylation, and primary DNA sequence variation such as single nucleotide polymorphisms (SNPs) could potentially be used as urinary prostate cancer biomarkers.^4^, ^7^, ^55^ Furthermore, proteomics has emerged as an attractive platform for discovering new biomarkers, opening up new avenues for understanding the prevalence and progression of urologic malignancies.^45^, ^46^, ^75^ As this review represents a selection of highlighted work that can potentially signify the future trends of nano‐diagnostics in PCa, niche urine biomarker players, including the cancer‐specific changes in urinary RNAs^121^ and urinary metabolites,^122^ will not be fully elucidated.

#### 3.2.1 RNA‐based markers

Urine contains a variety of prostate cancer‐related RNAs, including long noncoding RNAs (Inc RNAs) and micro‐RNAs (miRNAs), which has made RNA‐based biomarkers the most widely studied subtype of urine genetic biomarkers.^123^, ^124^, ^125^

PCA3, formerly known as differential display code 3 (DD3), was found in radical prostatectomy specimens in 1999 by comparing mRNA expression patterns of tumors with neighboring non‐neoplastic tissues.^117^, ^126^, ^127^, ^128^ PCA3 is a noncoding RNA biomarker that can be collected and detected in urine, and it has been found to be greatly overexpressed in 95% of PCa tissue when compared to normal prostate tissue from the same patient. Hessels *et al.* created a sensitive reverse‐transcriptase polymerase chain reaction (RT‐PCR) technology in 2003 to confirm that PCA3 mRNA expression levels in PCa tissue were 66‐fold higher than in normal prostate tissue.^127^ Furthermore, no expression of PCA3 was found in other normal human tissues, tumors originating from the breast, cervix, or testis, or cell lines originating from the bladder, kidney, or ovarian cancers, confirming PCA3's prostate‐specificity. PCA3 appears to be less affected by patient age, prostate volume, inflammation, or past biopsies than PSA.

PSA‐based screening techniques have poor sensitivity and low specificity, generating misleading results that lead to over‐diagnosis and over‐treatment, PCA3 as a next‐generation biomarker therefore has been incorporated into initiatives to improve PCa diagnostics.^127^ The PCA3 measurement is superior to blood PSA for predicting biopsy outcome, according to numerous studies, especially when considering the necessity for a repeat biopsy in persistent high‐risk men who had a negative first biopsy. PCA3 levels in radical prostatectomy specimens were similarly linked to cancer aggressiveness in preliminary studies. Although the PCA3 biomarker's potential in some diagnostic circumstances is encouraging, PCA3 as a stand‐alone test usually leads to a high rate of underdiagnosis of high‐grade PCa disease. To resolve this issue, combining PCA3 with other possible molecular markers is evaluated to yield better results for clinicians to make informed decisions.^54^

Genetic rearrangements that result in the production of fusion genes have been recognized as important events in cancer formation for more than 30 years. The fusion of the androgen‐related transmembrane protease serine 2 gene with the ETS‐related gene (TMPRSS2‐ERG), which accounts for 90% of prostate cancer fusions, has been linked to aggressive tumors with a fatal phenotype and a poor prognosis.^129^, ^130^ TMPRSS2‐ERG fusions, first found by Tomlins *et al.* in 2005, are specific for prostate cancer and could be detected in patients' urine.^129^

Hessels *et al.* investigated these fusion transcripts in the urine sediments of 108 prostate cancer cases after the digital rectal examination to further evaluate the possibility of TMPRSS2‐ERG fusions in the early identification of PCa (DRE).^131^ TMPRSS2‐ERG fusion transcripts can be detected with a sensitivity of 37% and a specificity of 93%, according to the findings. In addition, the presence of TMPRSS2–ERG fusion transcripts in prostate biopsies are unrelated to the Gleason score. However, it is questionable if the presence of a TMPRSS2‐ERG fusion constitutes a predictive biomarker: Nearly 50% of prostate cancers lack TMPRSS2‐ERG. As a result, it's most commonly used in multiplexed assays alongside other particular biomarkers such as PCA3 and KLK3. Research of over 1300 people found that combining PCA3 and TMPRSS2‐ERG measures in urine successfully identified groups with markedly different risks of cancer, high‐grade cancer, and clinically significant cancer on biopsy, exceeding serum PSA and single TMPRSS2‐ERG for prostate cancer diagnosis, decreasing wasteful biopsy and overdiagnosis, and laying the groundwork for precision prostate cancer care.^130^

MicroRNAs (miRNAs) have ushered in a new age in molecular biology. miRNAs are naturally occurring small noncoding RNAs (approx. 20–22 nucleotides) that influence one‐third of gene expression posttranscriptionally and are thus being investigated as potential clinical diagnostic biomarkers for a variety of malignancies, including PCa. More than 2000 miRNAs have now been found in humans, and more than 200 of these are highly stable and have been tested in urine samples.^132^, ^133^ The utility of urine miRNAs for improving the clinical analysis of prostate malignancies has been validated, either alone or in combination with clinical indicators. A selected group of miRNAs, including miR‐141, miR‐21, miR‐200b, miR‐221, and miR‐375, have been the most extensively studied miRNAs for clinical PCa analysis. According to Koppers‐Lalic *et al.*, both miR‐21 and miR‐375 levels were lower in PCa patients' urine.^118^ However, because of the methodological differences in the studies, particularly in the examination of urine samples, there is a risk of ambiguous outcomes. In general, urine‐based investigations did not consider the possibility that urine samples following DRE could turn dipstick‐positive for hematuria, as has been found in 30%–40% of samples. As a result, studies using hemolysis‐affected miRNAs as markers are likely to yield skewed results. MacLellan *et al.* discovered that miRNAs were shown to be significantly upregulated (by a factor of three) in hemolyzed samples, indicating that hemolysis should be considered when assessing miRNAs in urine, particularly after DRE, to prevent biasing the results.^134^ To summarize, the preliminary use of urinary miRNAs as predictive biomarkers for PCa is still in its early stages, and more appropriately designed studies are needed to determine whether miRNA biomarkers provide additional information beyond conventional strategies for predicting biopsy outcome or disease progression.

#### 3.2.2 DNA‐based markers

Of all epigenetic modifications, DNA methylation has become the most intensely studied epigenetic modification in mammals. The covalent addition of a methyl group occurs generally in cytosine within cytosine‐guanine dinucleotide (CpG) islands.^135^, ^136^ In particular, hypermethylation that represses transcription of associated gene promoter regions leading to gene silencing has been most extensively studied because of its critical role in human carcinogenesis.^137^, ^138^ Detected with the use of a polymerase chain reaction (PCR) on urinary cells, several DNA methylation genetic markers have been investigated increasingly in prostate cancer.

Initial investigations found that assays for identifying GSTP1 hypermethylation in urine samples had an excellent specificity (ranging from 93% to 100%) and moderate sensitivity (ranging from 21.4% to 38.9%) for diagnosing PCa but that this could be improved to 80% in combination with prostate massage (DRE). GSTP1 methylation study of post‐biopsy urine specimens was performed by Mark et al. A total of 7 of 18 (39%) patients with prostate cancer (as determined by the first biopsy) had detectable GSTP1 methylation in their urine (58% sensitivity among valid cases).^139^, ^140^ Meanwhile, aberrant GSTP1 methylation was found in the urine of 7 of 21 (33%) patients who had no evidence of cancer on biopsy and 4 of 6 (67%) patients who had atypia or high‐grade prostatic intraepithelial neoplasia. Paul *et al* discovered GSTP1 methylation in 22 of 28 (79%) prostate cancers in another investigation. The associated urine‐sediment DNA was positive for GSTP1 methylation in 6 of 22 (27%) patients, confirming the presence of neoplastic DNA in the urine.^138^, ^141^, ^142^

Several other urine DNA indicators have been studied for prostate cancer in addition to methylation markers. In urine samples from prostate cancer patients, Chiou *et al.* found a greater quantity of 8‐hydroxydeoxyguanosine (8‐OHdG) than in normal control groups. Genetic markers (p14ARF, p15INK4b, and p16INK4a) were variably elevated during prostate cancer progression, according to Zhang *et al.* The RAS‐association domain family, often known as RASSF, has been linked to the development of prostate cancer.^143^ Till now, however, a primary limitation in applying methylation‐specific PCR‐based approaches for early detection of PCa is still the scarce quantity of ctDNA in plasma in patients with early‐stage cancers.

#### 3.2.3 Protein‐based markers

Proteomic studies have sparked a new era in the search for noninvasive cancer biomarkers. The analysis of secreted proteins in the urine is promising to detect changes early in the course of PCa and PCa progression.^46^, ^47^, ^144^ Importantly, urine rarely undergoes proteolytic breakdown after collection. Because urine has a number of advantages for proteome research, including noninvasiveness, convenience of sample collection, and lower interfering chemicals, it is paving the way for early detection and active surveillance of urologic cancers.

Apart from serum, PSA is found in urine, which has been linked to disease recurrence.^16^ Patients with prostate cancer and those with benign prostatic hyperplasia had similar urinary PSA levels, but the urinary to serum PSA ratio was considerably different, according to a study of 170 men.^145^ Another study reveals that when the serum PSA level falls between 2.5 and 10.0 ng/ml, the urine to serum PSA ratio could be a useful tool for detecting prostate cancer.^146^

Preliminary investigations for the diagnosis of prostate cancer have looked at a variety of additional urine protein indicators. Calgranulin B (MRP‐14) in urine has emerged as a possible marker of prostate cancer, first identified by Rehman *et al.* ^45^ A gel‐based proteomic method was used to evaluate 12 urine samples from patients with prostate cancer or benign prostatic hyperplasia. In a trial of 106 males, the researchers were able to distinguish cancer from benign hypertrophy with 67.4% sensitivity and 71.2% specificity using urinary calgranulin B. Muller *et al.*, on the other hand, found no link between urine MRP‐14 and prostate cancer^147^ In patients with prostate cancer, urinary‐MSMB was lower than in individuals with benign prostatic diseases. The sensitivity of prostate cancer diagnosis was enhanced when serum PSA was included.^148^ CAV1 and CAV2 levels in the blood were higher in CRPC patients than in non‐castration‐resistant patients.^149^

Prostate cancer and benign prostatic diseases could be distinguished by a signature of 12 urine peptides. This signature increased detection when combined with age, free, and total PSA.^150^ In urine from prostate cancer patients, delta‐catenin immune scores were considerably higher than in control samples. When an immunological score cutoff of 45 was used, 87.5% sensitivity and 83.3% specificity were found.^151^ Besides, other assorted types of proteinic biomarkers include glypican‐1 (GPC‐1), leptin, osteopontin (OPN), and vascular endothelial growth factor (VEGF), indicating aggressive PCa when levels increased.^152^ Recently, niche protein‐based markers, for example, the cancer‐testis antigens (CTAs)^153^ and the urokinase plasminogen activator (uPA),^154^ were also reported for PCa analysis. Although many proteinic candidates produced promising results in the laboratory, several pitfalls, including deficient sensitivity, poor specificity, and low predictive value, make them less than desirable in a practical patient setting.

## 4 NANO DIAGNOSTICS

Early identification of malignant cell development and metastasis are the goals of PCa diagnostic techniques. The use of positron emission tomography (PET), magnetic resonance imaging (MRI), computed tomography (CT), and ultrasound are the standard methods to diagnose PCa. However, these imaging technologies lack the capability to provide information about PCa types and stages, making it difficult to gain a complete assessment of the disease and selection of treatment.^87^ As a result, there is a high demand for accurate PCa screening technologies.

**FIGURE 5:**
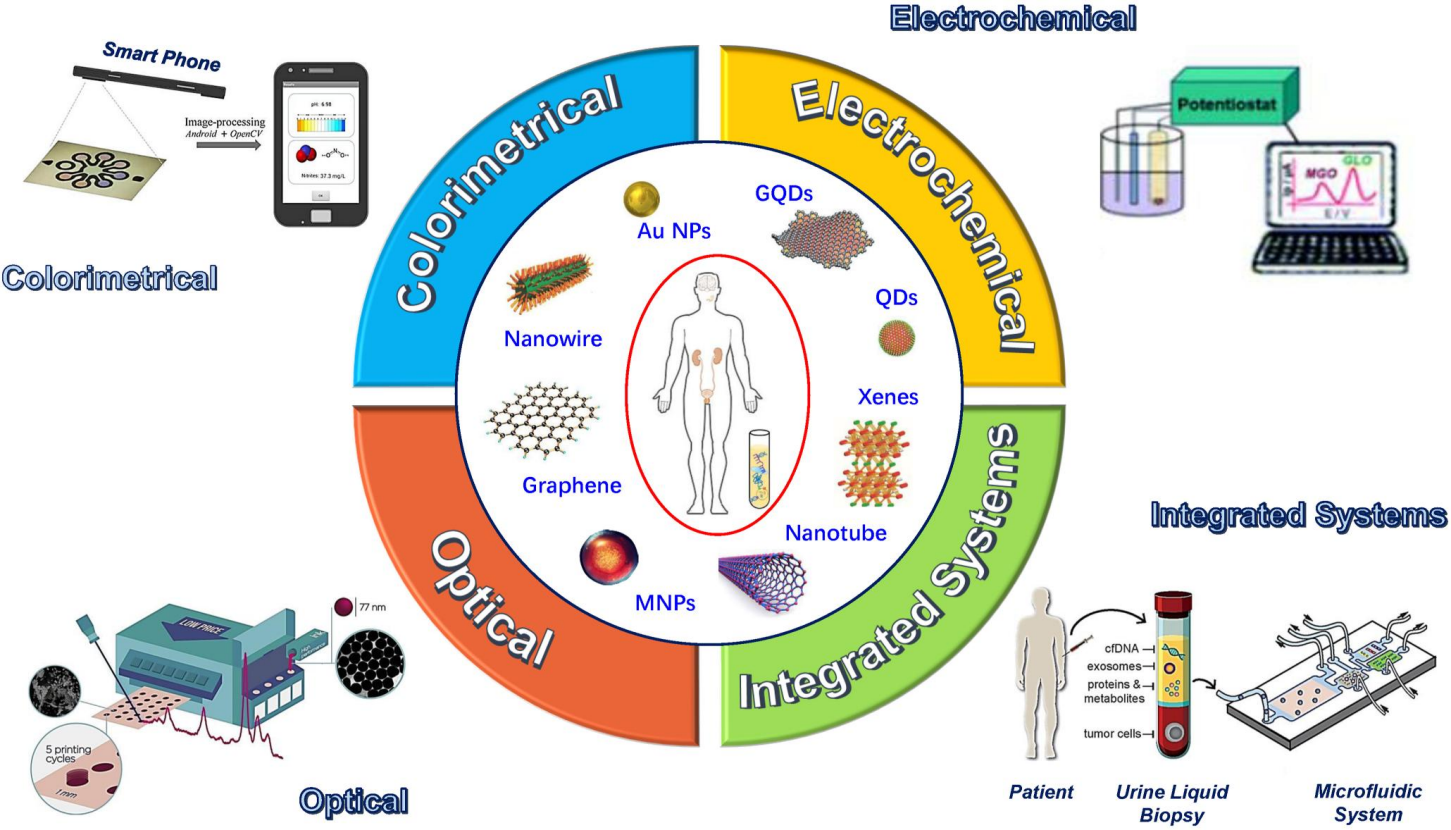
A plethora of emerging nanomaterials backed with novel analyzing platforms, including colorimetrical, electrochemical, optical, and integrated all‐in‐one systems, blazing a trail for the precision management of prostate cancer.

To address the shortcomings of existing PCa diagnostic techniques, the advances in materials science and nanotechnology provide exciting new diagnostic opportunities for PCa. Nano‐diagnostics, the combination materials and technological paradigm, refers to a wide range of clinical diagnostic procedures that use nanotechnology to enable highly parallelized analyses, reduce time, cost, and reagent and sample consumption, and improve sensitivity and specificity, resulting in comprehensive, accurate, and economical cancer early detection,^25^, ^61^, ^62^, ^155^ as schematically shown in Figure 5.

### 4.1 Nanomaterials: Small matters

As a pivotal part of nano‐diagnostics, nanomaterials offer great technological advantages. In the past few decades, the application of nanomaterials in cancer diagnosis and monitoring has garnered increasing attention.^156^, ^157^ Nanomaterials are objects and structures with dimensions ranging from 1 to 100 nm, and because of their small size, they exhibit physico‐chemical properties and functionalities that differ from those exhibited at the micro/macro scale materials, as well as a higher surface area to volume ratio.^158^, ^159^, ^160^ Constant advances over the past decade have made it possible to manufacture nanomaterials in different shapes, surface chemistry, and elemental composition. Nanomaterials have been successfully deployed for diagnostics where these systems provided new eyes to study cancer on other critical diseases.^161^, ^162^, ^163^ In this section, we will discuss several promising nanomaterials for the diagnosis of PCa, including nanoparticles, quantum dots, graphene, and carbon nanotubes.

#### 4.1.1 Nanoparticles (NPs)

**FIGURE 6:**
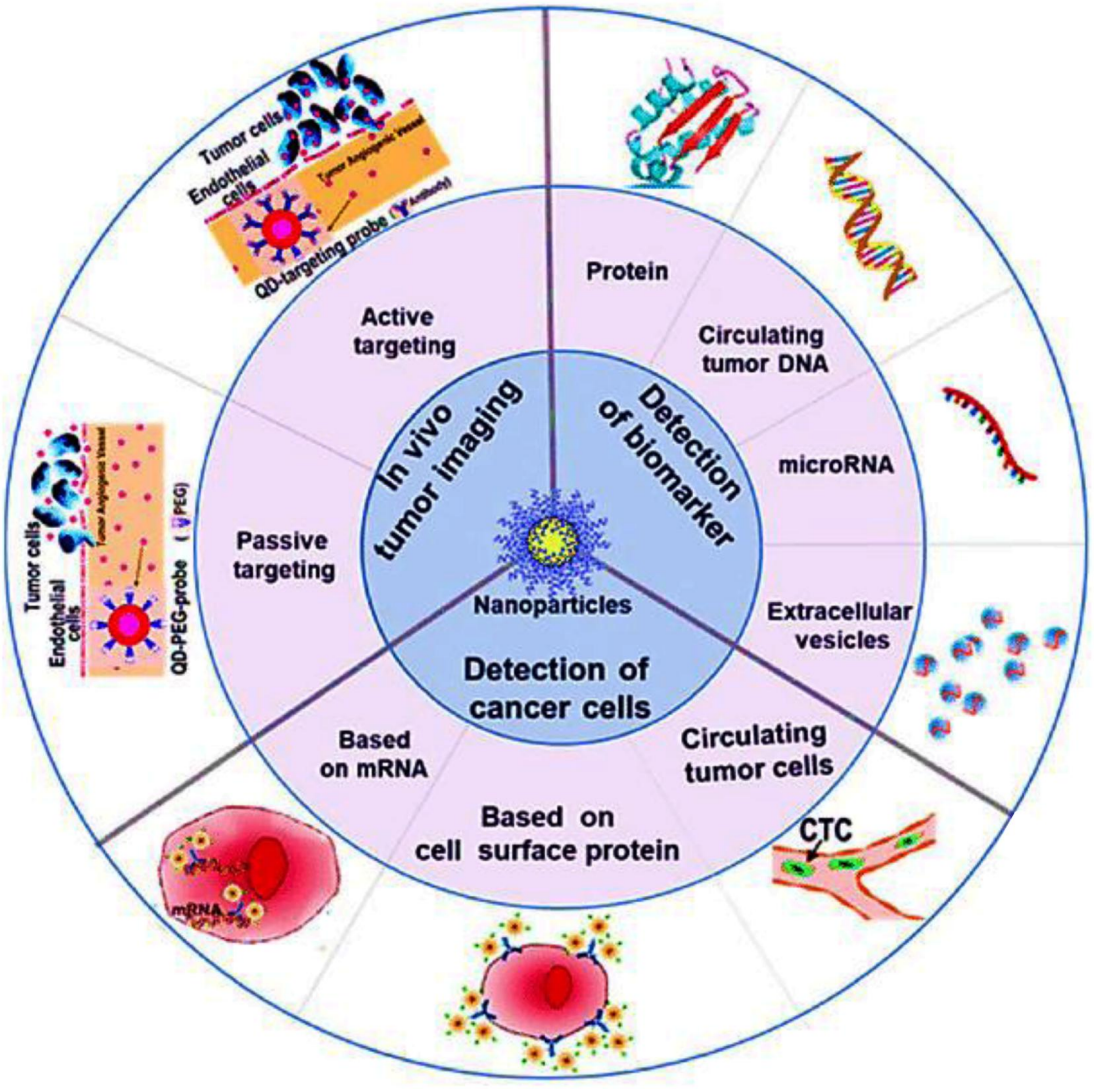
Schematic illustration of nanoparticle applications in cancer diagnosis. Reproduced under terms of the CC‐BY license.^64^ Copyright 2019, The Authors, published by Springer Nature Limited.

A plethora of nanoparticles, mainly with diameters several nm to several hundred nm, have been created and investigated for their potential use as diagnostic agents throughout the previous few decades, see Figure 6. Gold nanoparticles, magnetic nanoparticles, and polymeric nanoparticles are among the possible nanomaterial forms that have intrinsic features that influence their biodistribution, target site accumulation, and removal. Particle size and charge, core and surface properties, shape and flexibility, as well as multivalency and controlled synthesis, are the most important properties of nanoparticles because they determine the nanoparticle's distribution, targeting ability, and toxicity.^164^

##### Gold Nanoparticles (AuNPs)

In biomedicine, gold‐based nanoparticles are arguably the most studied colloidal nanoparticles. Gold nanoparticles have piqued attention in the management of cancer sickness during the last decade because of their combined various diagnostic capabilities. The unusual chemical‐physical features of AuNPs, notably including superior biocompatibility and excellent stability, which allow for the customization of numerous diagnostic functions, are largely responsible for their multifunctionality.^165^ Despite advancements in prostate cancer diagnosis, it is critical to continue to improve existing diagnostic processes to detect the tumor at an early stage. The serum biomarker PSA is one of the most reliable diagnostic and prognostic markers for PCa diagnosis. Typically, conventional PSA blood tests have a detection limit of 4 ng/ml. The benefits provided by AuNPs highlight the need for increased detection sensitivity.

Because of their capacity to be employed as tags to allow for increased sensitivity, the emergence of AuNPs has made significant progress possible. Barbosa *et al.* recently discovered a new approach for attaining optimal one‐step quantification of PSA utilizing silver‐enhanced gold nanoparticles linked to anti‐PSA antibodies and carbon nanoparticles.^165^ They devised a new optical and low‐cost detection approach that used a microcapillary film (MCF) as a platform for immunoassays. PSA was successfully quantified, with a dynamic range of 10–100 ng/ml. Later, Rodriguez *et al.* went on to describe a porous silicon electrode platform that was connected to 100 nm gold nanoparticles (Au NPs) and coated with anti‐PSA antibodies. The PSA was firstly paired with a surface‐anchored antibody and then through a secondary antibody tethered to the Au NPs. As the Au NPs increased conductivity, the detection limit is further improved to 1 ng/ml PSA.^166^

##### Magnetic Nanoparticles (MNPs)

Magnetic nanoparticles, particularly magnetic iron oxide nanoparticles (MIONs), have shown to be one of the most successful inorganic nanomaterials for biological applications. Their unique nanoscale magnetic phenomena provide the ability to develop new clinical diagnostics that other nanomaterials cannot match.^167^, ^168^ MIONs can respond to an external magnetic field by exhibiting a variety of magnetic‐responsive behaviors for a variety of purposes. Eiamphungporn *et al.* have proposed an MNP‐based PCR combined with a colorimetric enzyme‐linked oligonucleotide test for urinary PCA3 detection.^128^, ^169^ In the test, the forward primer was labeled with MNP, and the reverse primer was labeled with dual biotin that combined with a horseradish peroxidase‐streptavidin detection system. The proposed approach demonstrated great analytical sensitivity and specificity, detecting PCA3 at femtogram level that was approximately 1000‐fold more sensitive than the conventional RT‐PCR. Furthermore, based on PCA3 levels in urine, this technique differentiated PCa patients from both healthy controls and benign prostatic hyperplasia (BPH) patients.

##### Up Conversion nanoparticles (UCNPs)

Rare‐earth up‐conversion nanoparticles (UCNPs) have attracted widespread attention among the many nanomaterials created in the last few decades. Long‐wavelength photons (such as near‐infrared light) are continually absorbed by UCNPs, which then radiate short‐wavelength photons via two‐photon or multiphoton processes. As a result, UCNPs have a number of benefits over typical organic fluorescent dyes for use in biological systems, including a large Stokes shift, weak photobleaching, low toxicity, and good colloidal stability, thus making them promising for use as integrated probes for cancer early detection.^152^, ^170^, ^171^

The Kanaras group, for example, demonstrated the successful implementation of a graphene oxide and up‐conversion nanoparticles (NPS) sensor technology for the selective detection of mRNA‐related oligonucleotide PCA3 for potential early diagnosis of PCa.^171^ The approach facilitated femtomolar detection sensitivity of PCA3 in patient plasma. Jin *et al.* recently described a single‐molecule sandwich immunoassay for diagnosing aggressive prostate cancer by visualizing single nanoparticles.^152^ Their assay used photo‐stable UCNPs as labels to detect four types of circulating antigens in blood circulation: GPC‐1, leptin, OPN, and VEGF, all of which have high serum concentrations and indicate aggressive PCa. A single UCNP doped with thousands of lanthanide ions can emit an anti‐Stokes' luminescence that is intense enough to be quantitatively visible under a wide‐field microscope. Limit of detection (LOD) of biomarkers can be determined by counting every streptavidin functionalized UCNP that is specially tagged on each sandwich immune complex. For GPC‐1, leptin, OPN, and VEGF, they attained LOD of 0.0123 ng/ml, 0.2711 ng/ml, 0.1238 ng/ml, and 0.0158 ng/ml, respectively. By monitoring minuscule concentration fluctuations in a panel of serum indicators, the developed single‐molecule assay demonstrates its potential in clinical use for prostate cancer detection.

#### 4.1.2 Quantum dots (QDs)

**FIGURE 7:**
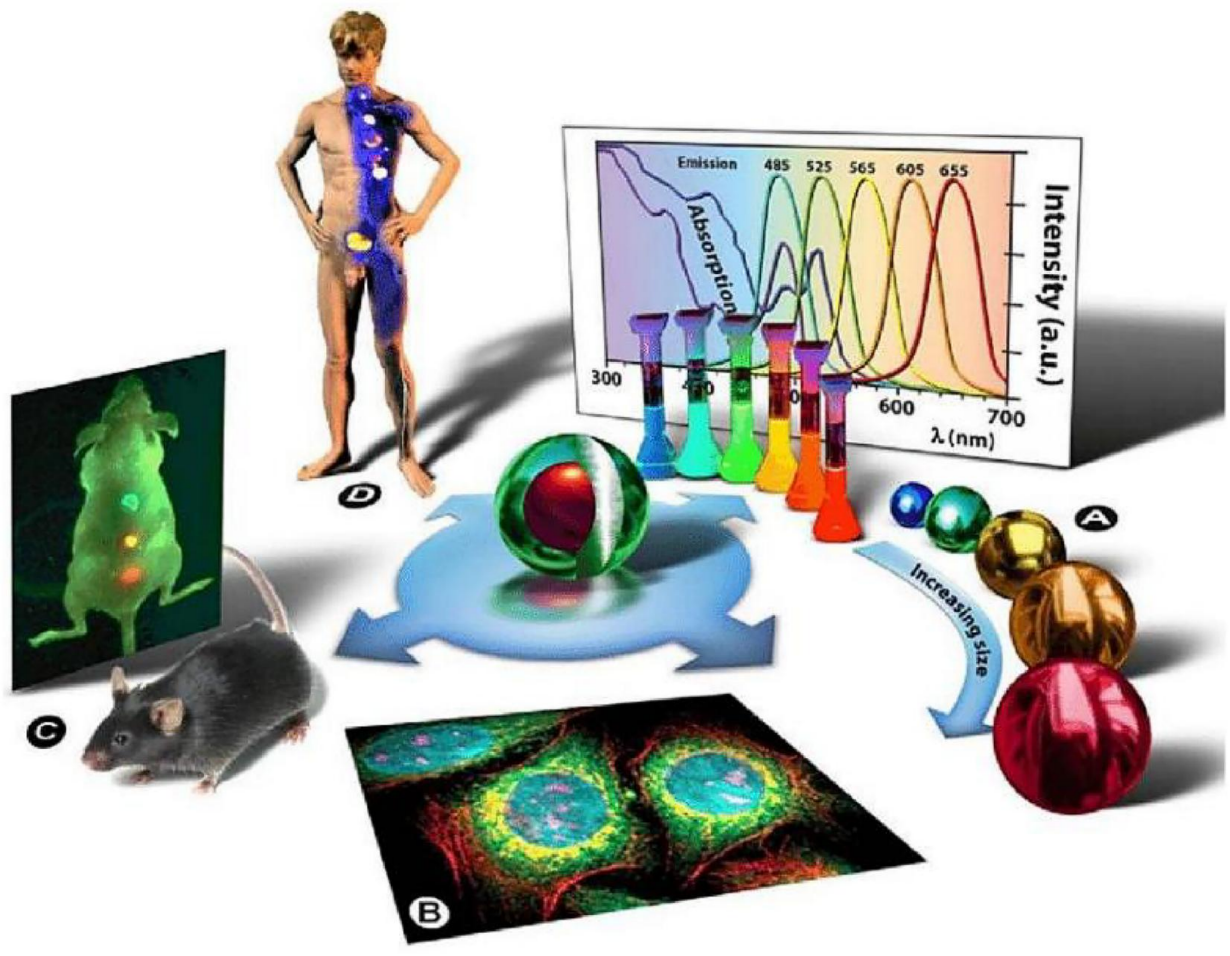
Quantum dots as multifunctional research and diagnostic tools for cancers. Reproduced under terms of the CC‐BY license.^177^ Copyright 2010, The Author, published by MDPI.

Quantum dots (QDs) are engineered fluorescent nanoparticles of ∼2–20 nm size with unique optical and electronic properties, such as high brightness, long‐term stability, simultaneous detection of multiple signals, and tunable emission spectra, making them appealing as barcodes for PCa diagnostics.^172^, ^173^ QDs also have well‐controllable surface chemistries for binding to targets such as antibodies, small‐molecule ligands, peptides, and nucleic acids, making them versatile biological probes. The integration of QD‐based nanotechnology and cancer biomarkers, in particular, could open up new avenues in clinical oncology, such as cancer diagnosis, treatment, prediction, and monitoring.^174^, ^175^ see Figure 7.

Colloidal semiconductor quantum dots (SQDs) have dominated much of the cancer detection research over the last few decades. These are nanometer‐sized crystals of semiconductor materials including CdSe, ZnS, InP, and PbSe that have stabilizing ligands attached to their surfaces to prevent aggregation. Because the supply of these elements in nature is limited, SQDs are costly. Graphene quantum dots (GQDs)—or carbon counterparts to SQDs—have emerged as promising alternatives, giving numerous interesting options for precision cancer diagnosis due to their availability, low cost, and biocompatibility.^176^

In preliminary evaluation and research, the PCA3 gene shows tremendous promise in the diagnosis of PCa when compared to the PSA test. Fluorescence‐based assays have gained popularity due to their rapid reaction, high sensitivity, nondestructive nature, and ability to monitor in real‐time. Huang's team created an NIR fluorescence enhanced satellite structure probe for sensitive and specific detection of PCA3 using DNA1‐Au nanorods (NRs) and DNA2‐Ag_2_S QDs.^178^ The optimal metal‐enhanced fluorescence (MEF) effect distance between Au NRs and Ag_2_S QDs is 8.16 nm, which is perfectly matched with the length of the PCA3 sequence. The composite probe can boost the luminescence performance of NIR Ag_2_S QDs at an acceptable distance by using the MEF effect. PCA3 can couple DNA1‐Au NRs with DNA2‐Ag_2_S via the base complementary pairing principle to form satellite probing structures. PCA3 may be detected specifically and sensitively (a low detection limit of 1.42 p.m.) using the built fluorescence‐enhanced NIR fluorescent probe. More crucially, the composite probe may be utilized to determine PCA3 levels in complicated systems such as cell lysates (PC‐3 and LNCap cell lysate).

The degree of expression of certain TMPRSS2: ERG fusion genes are linked to pathologic features of aggressive PCa and disease progression. Lee *et al.* proposed using oligonucleotide‐functionalized quantum dots and magnetic microparticles for optical detection of rearranged TMPRSS2: ERG fusion genes at low concentrations in buffer, urine, and prostate cancer cells (LNCaP and NCI‐H660 cell lines) to develop an assay for prostate cancer diagnosis.^179^ Maleimide‐functionalized CdSe QDs were tethered to the thiol‐terminated detection oligonucleotides that are complementary to distinct regions of TMPRSS2: ERG gene. With a wide detection range and a detection limit of 1 fM, their system was able to detect three different types of fusion genes. Using color‐coded oligonucleotides, they were able to detect several TMPRSS2: ERG fusion genes in a single test.

With the growing promise of QDs in clinical cancer research for individualized prostate cancer analysis, several other major challenges need to be addressed before clinical and translational uses, in addition to biosafety: I) In individual models or therapeutic regions, the synthesis, surface modification, and characterization of QDs should be standardized; II) to improve reproducibility, quality criteria, or standardizations for labeling biomolecules with QDs should be defined; III) the molecular information shown by QD‐based nanotechnology must be precisely obtained, quantitatively evaluated, and correctly understood.

#### 4.1.3 One dimensional nanostructured materials (1DNMS)

**FIGURE 8:**
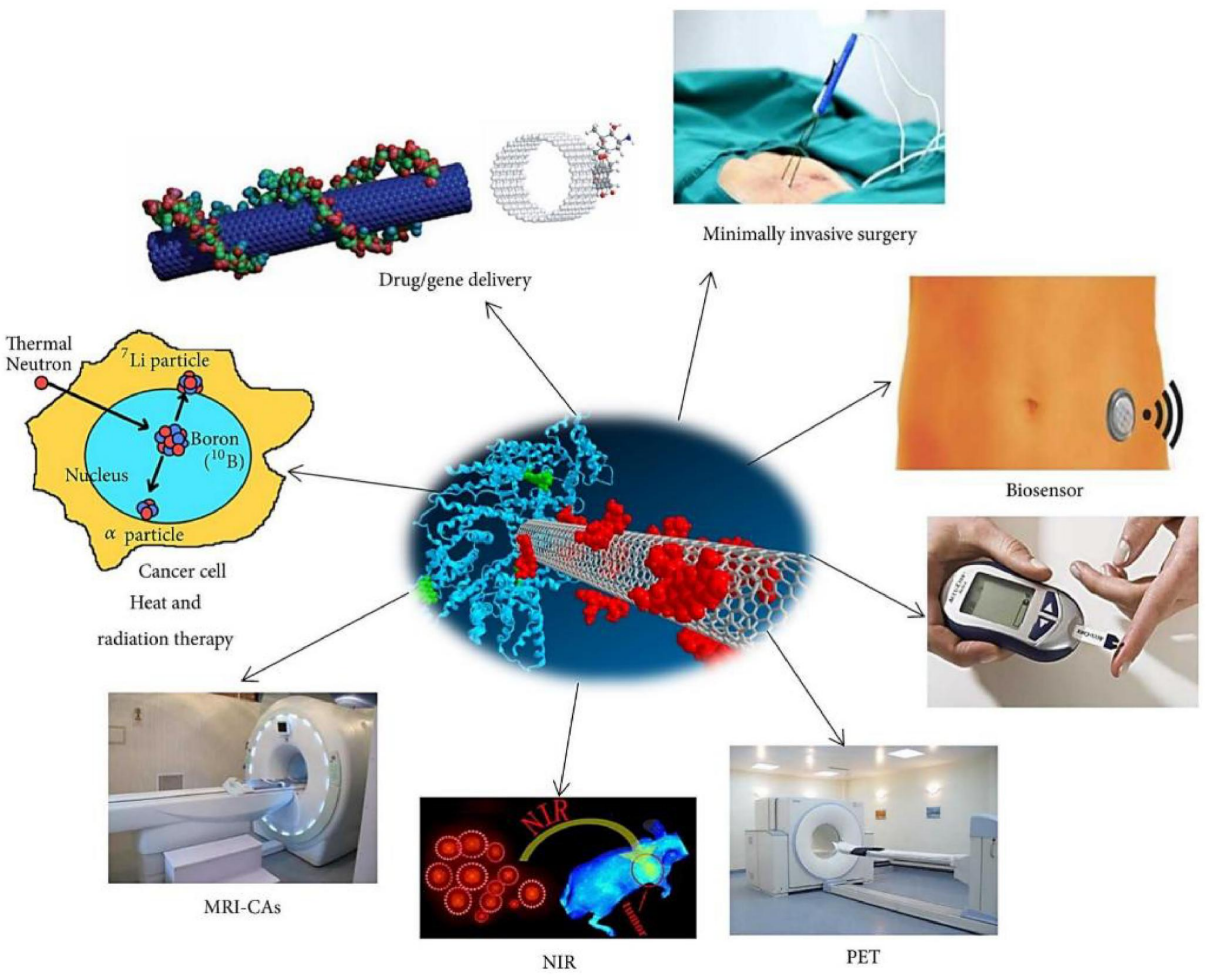
Biomedical applications of functionalized one‐dimensional materials in cancer diagnostic fields. Reproduced under terms of the CC‐BY license.^181^ Copyright 2017, The Authors, published by Hindawi.

During the last few years, the field of nanotube one‐dimensional (1D) nanostructure materials (1DNMS) have attracted increasing interest. It is widely agreed that 1DNMS are appropriate systems for researching the size‐ and dimension‐dependent functional qualities at the nanoscale and for exploring a variety of unique phenomena at the nanoscale.^180^, ^181^ Nanowires and nanotubes‐based 1DNMS, in particular, have shown promise as candidates for integration into portable devices that could change PCa detection and patient monitoring (Figure 8).

##### Nanowires‐based 1DNMS

Nano‐electrical sensors based on semiconductor nanowire field‐effect transistors (NW‐FETs) can provide high sensitivity, specificity, and a direct, label‐free electrical readout. They are also suited to large‐scale and high‐density integration, which is appealing. SiNW‐based sensors, in particular, have gotten a lot of attention because of their sensitivity in detecting biological and chemical species, as well as their mass‐manufacturability thanks to mature fabrication technologies, as demonstrated by Gao *et al.* ^182^

Promisingly, the nCounter Analysis System, which uses nanowire technology, detects CTAs in men with prostate cancer and correlates them with disease characteristics. The system binds captured mRNA hybridized‐reporter probe strands to the streptavadin‐coated nanowire surface via biotin molecules. Results show that men with Gleason score (GS) 8–10 disease had considerably higher RNA expression levels of the CTAs (CSAG2 and NOL4) than those with GS‐2 disease, according to a nanowire‐based test. In contrast, PAGE4 RNA expression was lower in men with GS 8–10 disease than in those with GS‐6 disease. This work shows that CTAs could be detected using a translatable Nanostring assay and that a collection of CTAs correlates with illness clinical features.^153^

The most prominent extracellular microRNAs (miRNAs) for the diagnosis of high‐risk PCa are miR141 and miR375. Using a plasmonic nanowire interstice (PNI) sensor, Yang *et al* achieved attomolar detection of miR141 and miR375 released from live PC cells.^183^ The surfaces of Au NWs were modified with probe LNAs through Au‐S chemical bonding. The probe LNA1 was modified for coupling the miR141 and the probe LNA2 was modified for analyzing the miR375. For all target miRNAs, this sensor had a low detection limit of 100 a.m. and a large dynamic range of 100 a.m. to 100 p.m. Furthermore, the PNI sensor was able to distinguish between a variety of single‐base mismatches in miRNAs. More crucially, the PNI sensor was able to detect extracellular miR141 and miR375 produced from living PC cell lines (LNCaP and PC‐3), demonstrating the sensor's diagnostic capacity for PCa.

##### Nanotubes‐based 1DNMS

Nanotubes‐based 1DNMS belong to the most widely studied nanomaterials for precision cancer care, with carbon nanotubes (CNTs) as an example. CNTs are made up of thin sheets of benzene ring carbons coiled up into a seamless tubular structure. They are divided into two types: single‐walled (SWNTs) and multi‐walled (MWNTs), which contain several concentric graphene sheets. Lerner *et al.* developed a novel detection method for osteopontin (OPN), a new biomarker for prostate cancer, by attaching a genetically engineered single‐chain variable fragment (scFv) protein with high binding affinity for OPN to a carbon nanotube field‐effect transistor (NT‐FET) to investigate the use of CNTs in PCa diagnosis.^184^ In the clinically relevant range, a concentration‐dependent rise in the source‐drain current is observed, with a detection limit of around 30 fM, which is three orders of magnitude lower than the current clinical standard‐ELISA immunoassays.

uPA is also a promising biomarker for determining the difference between aggressive, metastatic prostate cancer and indolent disease. Williams *et al.* developed a sensitive and selective sensor for the metastatic prostate cancer biomarker uPA using the optical characteristics of carbon nanotubes.^154^ The sensing mechanism of this structure was based on the modulations enabled by the passivation of hydrophobic SWCNT surface with bovine serum albumin (BSA). Through antibody conjugation, photoluminescent SWCNTs were designed to respond selectively to uPA, resulting in modification of the optical bandgap following analyte contact. The SWCNT sensor was demonstrated in a variety of human blood derivatives, paving the way for further research into the sensor's clinical application.

#### 4.1.4 Two dimensional nanostructured materials (2DNMS)

**FIGURE 9:**
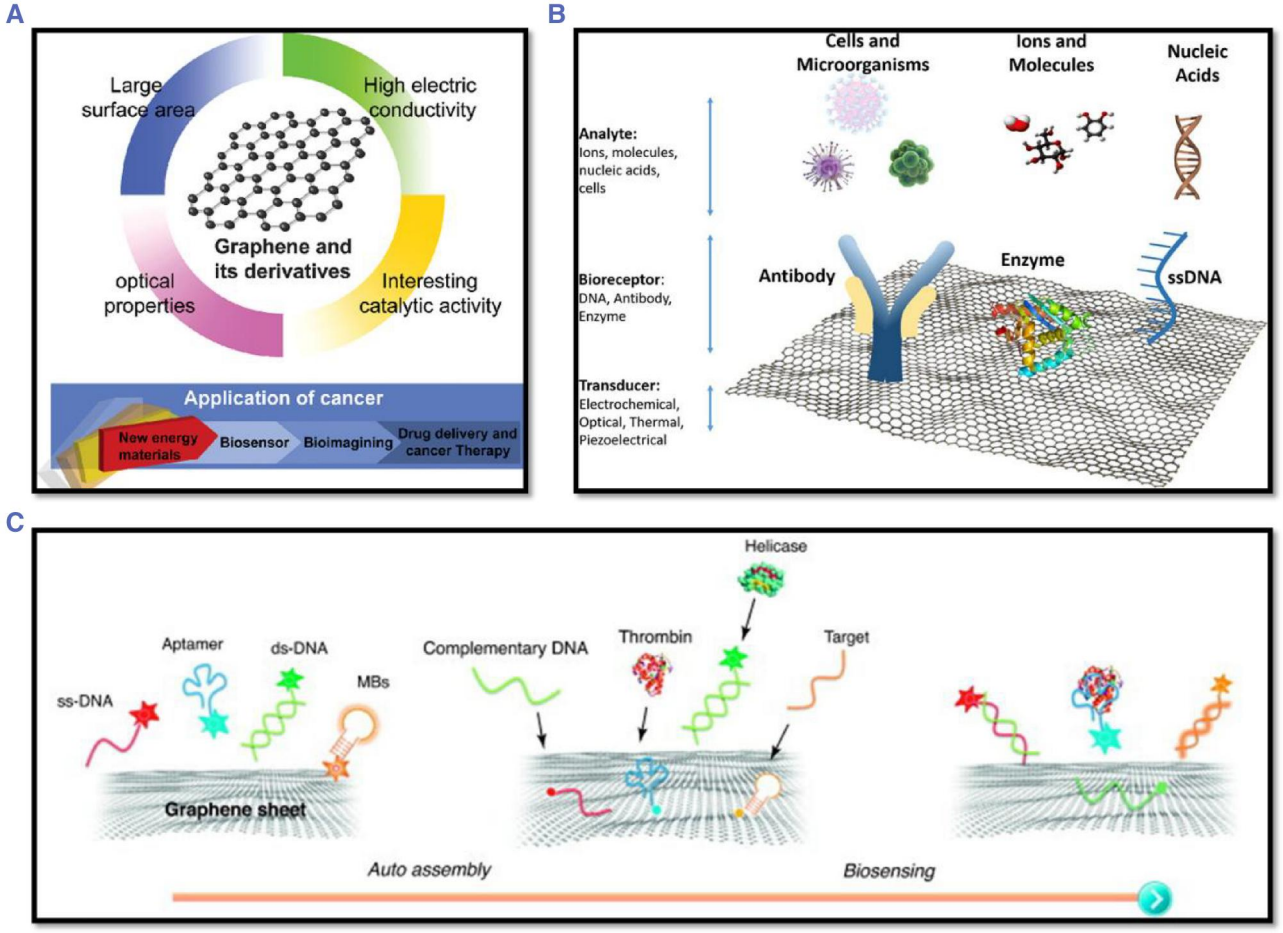
(A) The characteristics and potential applications of graphene and its derivatives. Reproduced with permission.^187^ Copyright 2018, American Scientific Publishers. (B) Various types of biosensors for cancer analysis based on a graphene platform. Reproduced under terms of the CC‐BY license.^188^ Copyright 2018, The Authors, published by Springer Nature Limited. (C) Principles of graphene‐based biosensors. ssDNA, aptamers, and MBs can be adsorbed onto the surfaces of graphene or graphene derivates (which also possess a planar surface and 2D structure). Reproduced with permission.^189^ Copyright 2011, Elsevier.

Two‐dimensional nanostructured materials (2DNMS) are a new type of nano‐formulation that has gotten a lot of attention in the scientific world. 2DNMS have unique physical properties, such as high electrical conductivity, strong optical scattering, strong light absorption, facile functionalization properties, high stability, and atomic thickness, and these features make 2DNMS promising for the sensing of PCa biomarkers, see Figure 9. 2DNMS based on graphene and mono‐elemental materials (Xenes) have recently been widely reported for cancer diagnosis.^185^, ^186^

##### Graphene‐based 2DNMS

Graphene is a carbon‐based thin film made up of single atoms that has unique properties. Graphene‐based 2DNMS, including graphene, graphene oxide (GO), and reduced graphene oxide (rGO), can be functionalized with aptamers to create conjugate materials that can be utilized to diagnose PCa. Multiplex detection of PSA, thrombin (TB), and hemagglutinin has been achieved using GO‐based aptasensors with different aptamers. Both DNA and RNA aptamers conjugated to the GO surface were shown to be adequately active. On a single chip, a GO aptasensor with two linear arrays detected TB and PSA simultaneously.^190^ The biological activity of an on‐chip GO aptasensor has been reported to be maintained even after the DNA aptamer was changed from TBA to PSAA. The selectivity for PSA detection was confirmed by the decrease in fluorescence intensity observed at lower concentrations. A dual‐modality biosensor coated with GO–ssDNA on a gold electrode and incorporating poly‐L‐lactide nanoparticles for signal amplification was used to detect VEGF and PSA in human serum using a similar strategy.^191^ A different notable targeted strategy made use of an amplified fluorescence method based on a quantum dot (QD)–aptamer/GO sensor that can detect PSA with great sensitivity.

##### Xenes based 2DNMS

The emergence of novel 2D Xenes (*e.g.,* borophene, gallenene, silicene, germanene, stanene, phosphorene, arsenene, antimonene, bismuthene, tellurene, and selenene) promises to break through the limitations in the practical applications of other 2D materials, demonstrating the remarkable potential for their applications in biosensors, bioimaging, therapeutic delivery, theragnostics, as well as in several other new bio‐applications.^186^

The MoS_2_ quencher was employed by Huang's team for sensitive detection of prostate cancer biomarkers.^192^ The measurement of DNA methyltransferase (MTase) activity was made possible by creating a MoS_2_‐affinity substrate DNA. It was made up of ssDNA as well as dsDNA. Through *van der Waals* interactions, the ssDNA permits the substrate DNA to adsorb on MoS_2_ nanosheets. As a result, the fluorophore connected to the end of the dsDNA counterpart's fluorescence was quenched. Simultaneously, the dsDNA contains a DNA adenosine methyltransferase‐specific motif (Dam). The methylation‐sensitive restriction endonuclease DpnI cleaves and releases the fluorophore when Dam methylates it. The methylation level is determined by recovering and quantifying fluorescence. Meanwhile, MoS_2_ nanosheets are used to detect DNA using hybridization chain reactions (HCRs). The 2D material is in charge of suppressing the background signal as well as controlling the detecting system's fluorescence emission. The presence or absence of target DNA is indicated by the “on” and “off” switches. The signal generated by target‐triggered HCRs between two hairpin probes is amplified, resulting in high DNA detection sensitivity.

### 4.2 Nanotechnologies: Miniaturized diagnostic platforms

Nanotechnology is a multidisciplinary field concerned with the design and engineering of items with a diameter of fewer than 500 nm (nm). Nanotechnology, according to the National Cancer Institute, presents an extraordinary, paradigm‐shifting chance to achieve significant advancements in cancer diagnostics by providing sensitive and quantitative approaches for cancer detection and diagnosis.^25^, ^61^, ^193^ In the last few decades, technique platforms, including colorimetric, electrochemical, optical, and integrated miniaturized systems, have been explored for improved cancer diagnosis.^194^, ^195^ Coupled with novel functional nanomaterials, as exemplified and discussed in the preceding section, these nano‐diagnostic platform developments raise exciting opportunities for personalized oncology in which genetic and protein biomarkers are used to diagnose cancers based on the molecular profiles of individual patients.^60^

#### 4.2.1 Colorimetric platform

**FIGURE 10:**
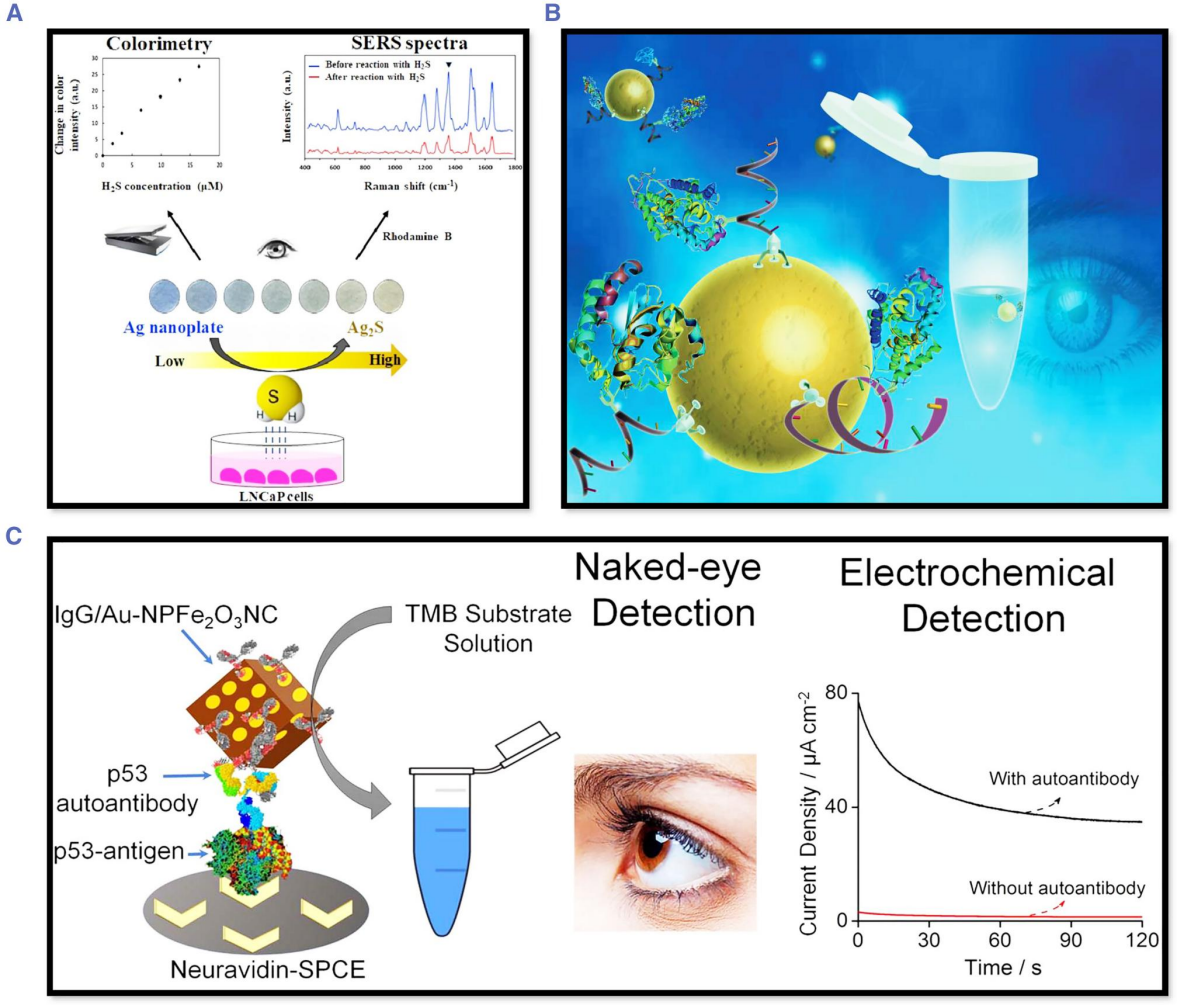
(A) Schematic illustration of the fabrication of Ag nanoplate‐coated H_2_S sensing paper and experimental design for dual‐mode colorimetric and SERS detection of H_2_S level in standard Na_2_S solutions and live LNCaP cells. Reproduced with permission.^199^ Copyright 2020, Elsevier. (B) Naked‐eye detection of isothermally amplified HOTAIR long noncoding RNA. Reproduced with permission.^200^ Copyright 2018, Royal Society of Chemistry. (C) Colorimetric and electrochemical detection of isothermally amplified HOTAIR long noncoding RNA. Reproduced with permission.^200^ Copyright 2018, Royal Society of Chemistry.

The colorimetric approach has garnered significant attention because of its ease, simplicity, and potential for point‐of‐care testing (Figure 10). Furthermore, costly, and intricate equipment is not required.^196^ In principle, the nanomaterials used can be classified into two groups: I) Noble metal nanoparticles are used as colorimetric substrates with localized surface plasmon resonance (LSPR) absorption, based on the NPs' aggregation and size/morphology transition and II) nanomaterials have intrinsic biological enzyme mimic activities, allowing them to serve as enzyme mimics and signal‐transduction tools, producing distinct color changes from the catalytic reaction product.^197^

Sarcosine is a novel biomarker for PCa. Yamkamon *et al.* devised an enzyme‐coupled colorimetric method for quantifying urine sarcosine.^169^ The assay detected sarcosine at concentrations as low as 0.7 μM without being hampered by ascorbic acid, glucose, or bilirubin. Later, Fu's group combined enzyme‐based sarcosine quantification with magnetic cross‐linked enzyme aggregates (MCLEAs) to present an enzyme‐based sarcosine quantification method.^198^ The method quantified sarcosine in urine, with a linear range of 0.3125–10 ng ml^−1^ and recoveries ranging from 87.50 to 97.75%, indicating that it has the potential to be a reliable method for sarcosine analysis in urine.

Due to their unique optical properties, gold nanoparticles (AuNPs) have been widely used for colorimetric detection. PCA3 was detected in urine samples with great sensitivity and specificity using a simple and rapid colorimetric test based on unmodified AuNPs and a thiol‐labeled PCR primer.^201^ In a salt solution, AuNPs typically aggregate (blue hue). DNA molecules strongly bind to the surface of AuNPs in the presence of thiol‐labeled PCR products, and the lengthy chains of DNA with numerous negative charges increase the electrostatic and steric repulsion among AuNPs, preventing salt‐induced aggregation (red color). Magnetic nanoparticles in combination with a colorimetric enzyme‐linked oligonucleotide assay detected relative PCA3 expression of PCa patients, which was substantially higher than that of BPH patients and healthy controls. The findings of the magnetic bead test were validated with qRT‐PCR.^128^ A colorimetric test for identifying TMPRSS2: ERG mRNA, a urinary gene fusion‐type biomarker for PCa, used a combination of isothermal RT‐RPA and magnetic TMB‐based colorimetric readout.^202^

The analytical performance of colorimetric nanotechnology platforms is dependent on high binding efficiency between the target and recognition element, the ability to transduce recognition events into colorimetric signals, and the anti‐interference capability toward nonspecific biomolecules. Further research is required to reduce nonspecific binding and achieve reliable and robust signal transduction in colorimetric nanotechnology platforms to improve their clinical translatability.

#### 4.2.2 Electrochemical platform

**FIGURE 11:**
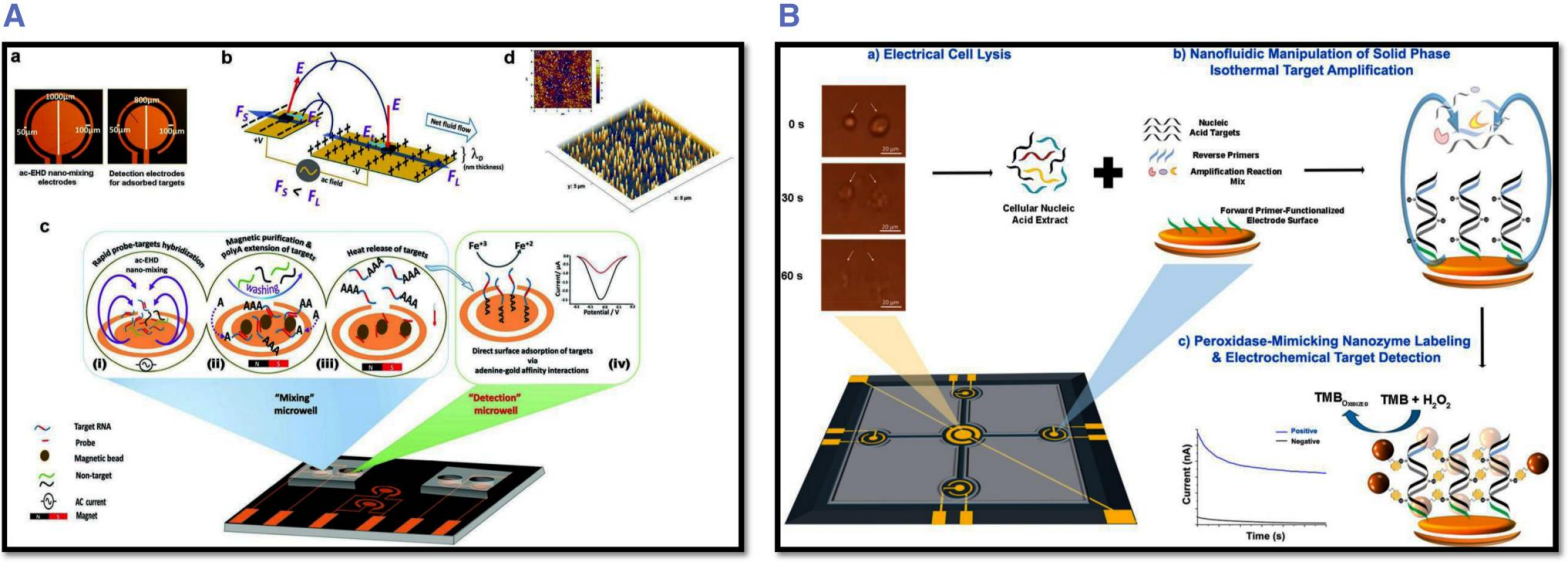
(A) Schematic of the analysis workflow. ac‐EHD nano‐mixing speeds up the probe‐target hybridization; streptavidin–magnetic beads were added to bind biotinylated probe‐target molecules for magnetic target purification, and subsequent polyA extensions of purified targets. Reproduced with permission.^205^ Copyright 2018, John Wiley and Sons. (B) Integrated biochip (with central lysis and four individual amplification/detection chambers linked by fluidic channels) for targeted gene analysis in liquid biopsies. Reproduced with permission.^206^ Copyright 2018, American Chemical Society.

In recent years, bioanalytical approaches have seen extraordinary expansion, owing in large part to the demand for faster, more sensitive, and portable (“point of care test‐POCT”) devices to detect biomarkers for clinical cancer detection. Electrochemical detection methods are advantageous since they are quick, easy, and inexpensive.^203^ The recent advances in electrochemical platforms have substantially improved our capability to detect low quantities of biomarkers present in the early stages of cancer by increasing the detection sensitivity. Importantly, the combination of electrochemical devices with nanomaterials such as gold nanoparticles, carbon nanotubes, magnetic particles, and quantum dots allows for the multiplexing of numerous cancer biomarkers. This section will discuss recent advances in the development of electrochemical platforms for the next generation of PCa diagnostics, with an emphasis on opportunities for further improvement for diagnostics, see Figure 11.

To electrochemically detect a biomarker, it needs to undergo or be part of a redox reaction that can be measured by a change in current, potential, or resistance. In label‐free electrochemical biosensing, the biomarker interaction with the sensor surface causes such a change. If the biomarker is not electrochemically active, a label can be added to the detection system to measure the biomarker. For instance, Nabok *et al.* labeled aptamers with electrochemically active redox groups. The redox label is brought closer to the electrode because of conformational change, resulting in an increase in charge transfer. They prepared a unique CG‐3 RNA‐based aptamer that is specific to 277 nucleotides of the PCA3 transcript.^204^ The aptamer is ferrocene‐labeled, allowing the identification of a 277‐nt lncRNA segment from PCA3. However, the effects of changes in secondary structures of both the PCA3 and the aptamer during binding remain elusive. Their findings are a first step toward the long‐term goal of establishing a unique, accurate, simple, and cost‐effective PCa diagnostic tool.

For PCa analysis, electrochemical sensors with carbon printed electrodes or quartz coated with layer‐by‐layer (LbL) films containing gold nanoparticles and chondroitin sulfate, as well as a layer of a complementary DNA sequence (PCA3 probe), have recently been developed.^207^ Electrochemical impedance spectroscopy showed the highest sensitivity, with a detection limit of 83 p.m. in PCA3 solutions, whereas cyclic voltammetry and UV–vis spectroscopy had detection limits of 2000 and 900 p.m., respectively. Electrochemical sensors based on 2D MXenes have also been developed for PCa detection.^208^ The enzyme immobilization support Ti_3_C_2_TX MXene was utilized to build a biosensor for sensitive detection of sarcosine, a possible PCa biomarker found in urine. The amperometric measurement of H_2_O_2_ produced during the enzymatic process was used to indirectly quantify sarcosine. In the concentration range of 0.02–5 nM, the sensitive nanostructured MXene‐based biosensor selectively detected sarcosine in urine.

More recently, Trau *et al.* developed a series of strategies for PCa analysis. Initially, they built a nanodevice to uniquely use alternating current electrohydrodynamic (ac‐EHD) forces to enhance probe–target hybridization prior to direct native RNA target detection, without probe‐target amplification or surface functionalization.^206^ Noninvasive screening of PCa RNA biomarkers in patient urine samples was performed to exemplify clinical applicability. A strong correlation between multi‐RNA‐type expression and aggressive PCa was demonstrated. Later, they reported a biochip system that performs an entire workflow of integrated circulating tumor nucleic acids (ctNA) target analysis from urine.^205^ They successfully demonstrated the multifunctionality of this integrated electrochemical biochip for cancer risk prediction and cancer relapse monitoring. Such a miniaturized system has great potential for creating new cancer screening protocols that are of high clinical need.

#### 4.2.3 Optical platforms

**FIGURE 12:**
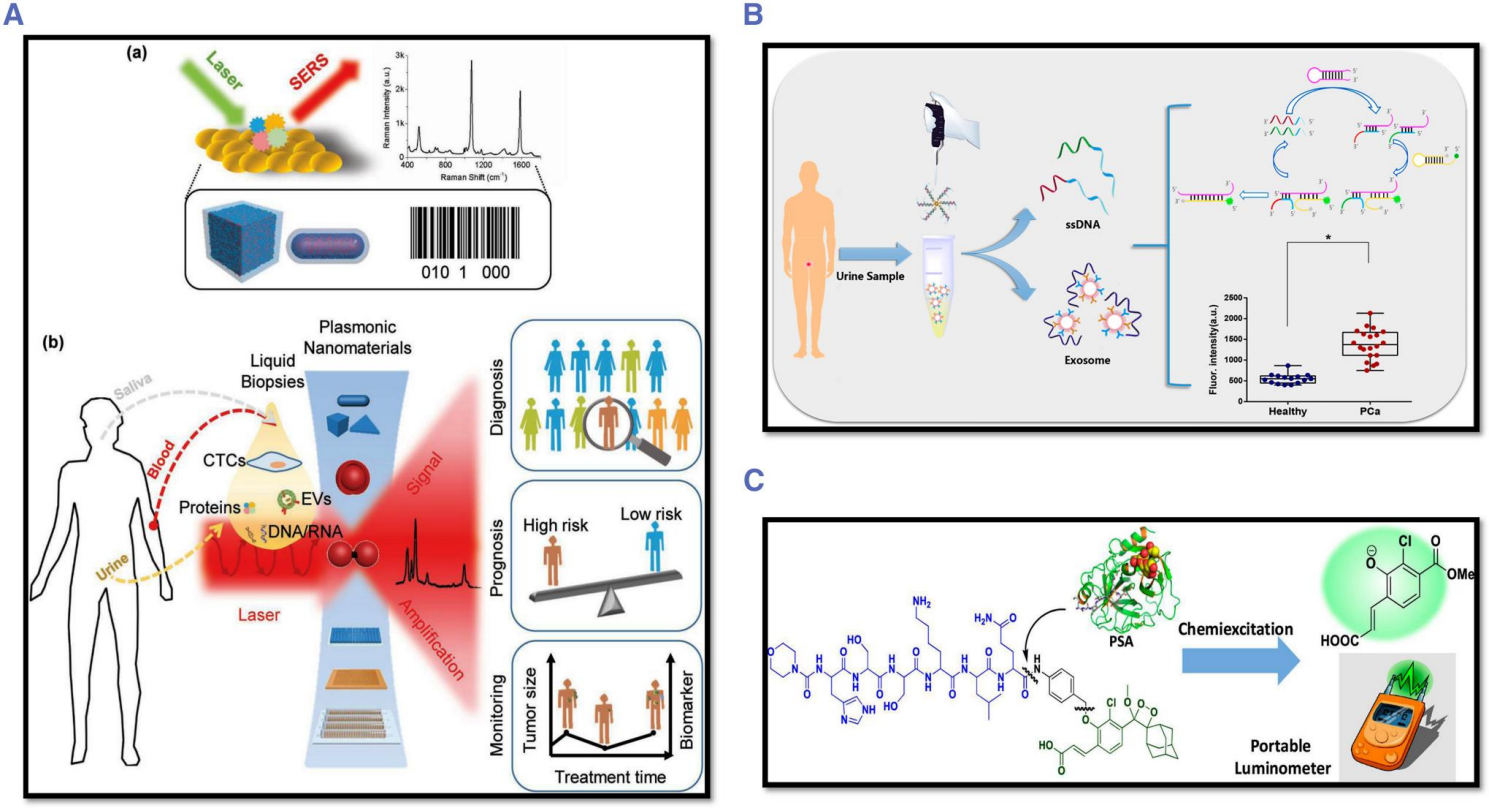
(A) Enhanced Raman scattering of molecules on or near the surface of plasmonic nanomaterials provides fingerprint information of molecules and could be used as a barcode signal in SERS measurements. Reproduced under terms of the CC‐BY license.^210^ Copyright 2019, The Authors, published by John Wiley and Sons. (B) A dual‐functional platform composed of superparamagnetic conjunctions and molecular beacons (SMC‐MB) of urinary exosomes for prostate cancer diagnosis. Reproduced with permission.^211^ Copyright 2019, American Chemical Society. (C) Robust chemiluminescence probe for rapid detection of prostate‐specific antigen proteolytic activity. Reproduced under terms of the CC‐BY license.^212^ Copyright 2020, The Authors, published by American Chemical Society.

Optical spectroscopy is the study of the frequency dependence of electromagnetic radiation (light) absorption, emission, and scattering/reflection interactions with materials. These interactions are represented in a spectrum carrying fingerprinting information about the sample's structure and composition. Optical readings can be quickly processed based on the spectroscopic signature of biochemical ingredients to offer an objective diagnostic without any specialized intervention.^179^, ^209^ As a result, rather than visible or microscopic changes in cellular or tissue shape, optical platform‐based diagnosis depends on biochemical changes underlying the pathology. Approaches based on fluorescence, chemiluminescence, and surface‐enhanced Raman scattering (SERS) have shown tremendous promise for improved noninvasive detection of prostate tumors, owing to the advances in optical nanotechnologies, see Figure 12.

By reducing waiting periods for histological diagnosis, the prospective application of optical spectroscopy‐based technologies for routine clinical diagnosis would reduce the frequency of follow‐up clinic visits and patient worries. Optical technologies represent no known dangers to patients and could thus be a safe complement to conventional diagnostic procedures.

##### Fluorescence

When a molecule is lit with an excitation wavelength that falls within its absorption spectrum, it absorbs the energy and transitions from the ground to the excited state. By emitting light at specified emission wavelengths, the molecule can then relax from the excited state to the ground state. The fluorescence intensity measured at a fixed excitation wavelength throughout a variety of emission wavelengths can thus provide information on the fluorophore's molecular properties. Cancer diagnosis based on autofluorescence of naturally occurring fluorophores was established in the late 1970s.^213^ Fluorescence spectroscopy has been proven to be a reliable platform for precision oncology management.^213^

The Fujimoto group investigated the feasibility and clinical utility of photodynamic diagnostics (PDD) of prostate cancer utilizing 5‐aminolevulinic acid (5‐ALA) to assess shed prostate cancer cells in urine samples.^214^ Before the prostate biopsy, urine samples were collected. Protoporphyrin IX (PPIX) positive (presence of cells displaying simultaneous PPIX fluorescence) or PPIX negative (absence of cells demonstrating simultaneous PPIX fluorescence) urine specimens were treated with 5‐ALA and photographed using fluorescence microscopy (lack of cells emitting fluorescence). 60 of the 81 PCa patients tested positive for PPIX (sensitivity: 74.1%). Even though traditional diagnostic tests revealed 57 patients to be free of PCa, 17 of these at‐risk patients were found to be PPIX‐positive (specificity: 70.2%). Promisingly, PPIX–PDD was discovered to be more sensitive than DRE and transrectal ultrasound, and more specific than the PSA testing assay.

##### Chemiluminescence

The chemiluminescence (CL) platform, defined as the emission of light (ultraviolet, visible, or infrared) because of a chemical reaction without the use of an external light source, is a simple, low‐cost approach that offers excellent sensitivity without the use of a light or power source.^212^ The interaction of luminol with hydrogen peroxide in the presence of a peroxidase enzyme can produce CL signals. The coupling of CL with 3D‐printed microfluidic devices opens the possibility of automated, multiplexed detection of cancer biomarkers using a simple camera for signal acquisition.

Li *et al.*, for example, devised a dual‐labeled gold nanoparticle approach to enhance chemiluminescence for extremely sensitive prostate‐specific antigen ELISA (PSA).^215^ Gold nanoparticles were used as the marker (Ab*) in this study, which was labeled with horseradish peroxidase and anti‐prostate specific antigen–antibody. After PSA (antigen, Ag) was introduced into the system, Ab* and Ag formed an immunological complex (Ag–Ab*) by a noncompetitive immune response. The intensity of Ag‐Ab*'s chemiluminescence was then utilized to calculate PSA concentration. In the range of 0.25–10 ng/ml, the calibration curve demonstrated high linearity, with a detection limit of 0.092 ng/ml. This approach was offered as an alternative tool for PSA in clinical analysis.

##### Surface‐enhanced Raman scattering (SERS)

SERS, a type of nanotechnology‐based optical Raman spectroscopy, has benefits over classic assay detection methods such as fluorescence and chemiluminescence. Sensitivity, multiplexing, photostability of Raman reporters, and the capacity to detect biomarkers in blood and other biological matrices are some of the advantages SERS offers for PCa diagnostics. The Raman signal of the Raman reporter is enhanced 10^6^–10^14^ times in the presence of plasmonic structures such as Ag nanoparticles (AgNPs) or gold nanoparticles (NPs), while the autofluorescence background interference is reduced. SERS has been widely used in recent years to identify proteins, nucleic acids, and other molecular biomarkers to diagnose many malignancies with great accuracies, such as breast cancer, colon cancer, and bladder cancer.^216^, ^217^

Urine contains metabolites that are elevated in PCa. In a study by Gui *et al.*, urine samples were obtained from healthy people and patients with bladder or prostate cancer and Raman fingerprints ranging from 500 to 1800 cm^−1^ were acquired of these samples using silver nanoparticles.^217^ Principal component analysis and linear discriminant analysis were used to classify the spectra (PCA‐LDA). The results showed that the model's classification accuracy for healthy people, bladder cancer patients, and prostate cancer patients was 91.9%, and the test set's classification accuracy was 89%, indicating that SERS combined with the PCA‐LDA diagnostic algorithm could be used to detect and distinguish bladder cancer and prostate cancer through urine testing.

#### 4.2.4 Integrated sample‐to‐answer systems

**FIGURE 13:**
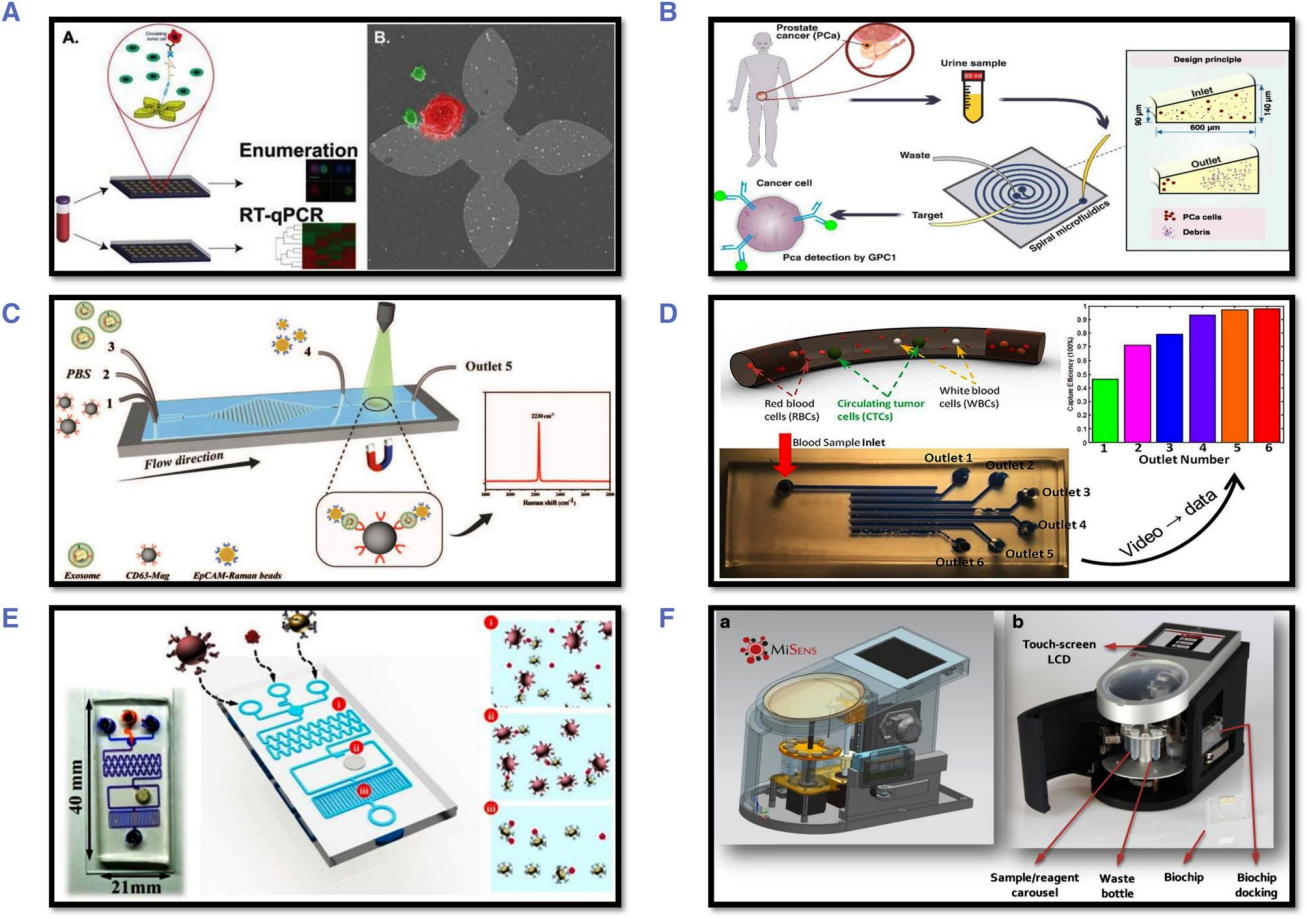
(A) Sample workflow of a graphene oxide chip enables isolation of prostate CTCs. Reproduced under terms of the CC‐BY license.^222^ Copyright 2019, The Authors, published by John Wiley and Sons. (B) Schematic representation of the workflow for PCa cell detection from the urine sample employing a spiral microfluidic chip. Reproduced under terms of the CC‐BY license.^84^ Copyright 2020, The Authors, published by MDPI. (C) Working principle of the microfluidic Raman chip for exosome capture and detection. Reproduced with permission.^223^ Copyright 2020, Royal Society of Chemistry. (D) A sequential size‐based microfluidic chip designed for the entrapment of prostate cancer circulating tumor cells. Reproduced with permission.^220^ Copyright 2018, American Chemical Society. (E) SERS‐based pump‐free microfluidic chip for highly sensitive immunoassay of prostate‐specific antigen biomarkers. Reproduced with permission.^224^ Copyright 2019, American Chemical Society. (F) Fully integrated and automated MiSens biosensor device. Reproduced with permission.^225^ Copyright 2016, Springer‐Verlag Berlin Heidelberg.

Ideally, cancer biomarker screening measurements would be performed with high accuracy, automation, and at a low cost at the point of treatment. Unfortunately, considering the point‐f‐care (POCT) scenarios, standard bench‐top laboratory procedures are not always suited to high‐throughput screening, which is responsible for large‐scale "‐omics” investigations. Intensive efforts have been made in recent decades to develop new and more reliable laboratory tests. Microfluidic technologies, also known as Lab‐on‐a‐Chip (LOC) or micro‐total analysis systems (TAS), provide significant hope in both point‐of‐care cancer biomarker measures and tailored diagnostic procedures, and have evolved to state‐of‐the‐art equipment for cancer research.^218^, ^219^ These platforms offer significant advantages in biological sample processing, throughput, reagent and sample consumption, assay time, and multiplexed detection. Microfluidic‐based technologies, in particular, have demonstrated the ability to increase molecular biomarker identification by providing sensitive and wide‐ranging measurements in a small format, making them a viable tool in the field of cancer diagnostics see Figure 13.

CTCs are widely recognized as an early sign of PCa diagnosis and severity. Currently, there are only a few commercially available products for capturing and enumerating CTCs, such as using the FDA‐approved CellSearch system. A sequential size‐based microfluidic high‐throughput entrapment chip for urine liquid biopsy analysis was developed to avoid the necessity for early‐stage CTC enrichment and entrapment for clinical diagnosis.^220^ When LNCaP‐C4‐2 prostate cancer cells were spiked in mouse whole blood at 50 cells/mL, this multiple‐row cancer cell entrapment device collected them with >95% effectiveness. Rzhevskiy et al. described a spiral microfluidic chip capable of separating PCa cells from PCa patients' urine with an efficiency of >85%.^84^

Exosomes, which are presumably discharged from the urinary system, have been discovered in the urine of PCa patients.^221^ The Cho group proposed a noninvasive liquid biopsy approach for analyzing the androgen‐receptor splice variant 7 (AR‐V7) in the RNA of urine‐derived EVs, a biomarker associated with CRPC.^41^ A lab‐on‐a‐disc with six independent nanofiltration units was used to isolate urine‐derived EVs, allowing six different samples to be processed at the same time. As a result, this method of quantifying AR‐V7 in urine EVs generated by a lab‐on‐a‐disc offers a potential approach to PCA liquid biopsy analysis.

In addition, for the study of protein PCa biomarkers, several elegantly built microfluidic systems were used. Corbera et al., for example, developed a new lab‐on‐a‐chip system for the precise analysis of spondin‐2 (SPON2), an extracellular matrix protein biomarker that is produced differently in malignant and noncancerous cells.^88^ A SERS‐based immunoassay that used a pump‐free microfluidic chip, as well as a prototype integrated lab‐on‐a‐chip‐based biosensor device, was also reported as a detection platform for prostate cancer diagnosis.^224^, ^225^

Despite the promise of integrated sample‐to‐answer systems for the precise detection of PCa, there are still various obstacles to overcome before these systems might be used at point‐of‐care for individualized PCa diagnosis. The fundamental issue is that most research has not been validated using relevant clinical samples, and only a small percentage of them have progressed to formal clinical trials. As a result, there is a significant gap between laboratory research and clinical use. Integration of an appropriate sample preparation technique into the current format of miniaturized devices is another hurdle to be mastered. We believe that increased research into quick and easy strategies for isolating biomarkers from crude biological material will effectively resolve this problem.

## 5 CHALLENGES AND PROSPECTS

The problems and demands involved with diagnosis and prognosis are growing in lockstep with the evolution of PCa therapeutic options. Despite the availability of molecular medicines to target specific genomic mutations, tumor heterogeneity remains a key hurdle in developing screening regimens based on individual genome analysis. CTCs, ctDNAs, and exosomes, for example, are at the heart of urine liquid biopsy diagnostics and have paved the way for new approaches to precision cancer care.^67^ In circumstances where primary tumors are difficult to biopsy, urine liquid biopsy could be a useful option. Furthermore, urinary liquid biopsy could help stratify patients and target screening modalities to those at higher risk, lowering side effects (such as mammography radiation) and healthcare expenditures.^226^

However, all these markers' clinical value will still need to be validated in the future. EVs and ctDNAs are more common and appear to be easier to study than CTCs, but they also pose technological problems. It is crucial to remember that the field of EVs and their potential for liquid biopsy is still in its early phases, and further research is needed to learn more about their role in oncogenesis and to clinically validate EVs for PCa analysis. While significant progress has been made in terms of biomarker isolation and detection strategies, key challenges related to the isolation of subpopulations of biomarkers or disease‐specific elements using a single technical platform must be addressed to facilitate clinical integration of nano‐diagnostic technologies. Furthermore, further investigation of disease‐specific biomarkers to determine their clinical potential and involvement in disease development should be performed for greater clinical validation.

In addition to the above, other hurdles must be overcome before liquid biopsies can fully realize their promise as true diagnostics for PCa management. A deeper understanding of the biology of EVs, ctDNA, and CTCs, in particular, can help establish a link between molecular profiles and the patient's physiological disease condition. While there has been evidence of a relationship between CTC numbers and ctDNA levels in certain cases, the fundamental biology behind this relationship is still unknown.^81^ The potential for ctDNA to be regarded as a full depiction of the cancer stage is one of several major issues involved with the application of liquid biopsy in clinical settings. Given the widespread use of circulating biomarkers such as ctDNA, CTCs, and EVs, it is intriguing to learn whether all metastases contribute to the release of these biomarkers into the bloodstream.^56^

The rapid development of nanomaterial‐based technologies, on the other hand, has demonstrated great potential for the identification of PCa‐relevant biomarkers. These nanodiagnostics technologies have been shown to offer refinements and improvements in PCa analysis through a large body of research and a range of investigations. Although the distinguished nanomaterials and advanced nanotechniques mentioned above have already been explored and exploited to make significant advances in prostate cancer detection, there are still a few significant challenges to overcome in moving these sensing platforms from the laboratory bench to the clinic bedside:

1. Simple sensing platforms without expensive and complicated devices are still needed for rapid and reliable PCa screening and diagnosis.
2. It is critical to avoid or minimize false positive or negative detection signals to achieve rational and valid output results in each nano‐diagnostics.
3. A simple and convenient system to amplify colorimetric signals with high sensitivity for early‐stage PCa screening is required.
4. Assembling components with various functions to produce the expected versatile platform with high selectivity necessitates more innovation.
5. Monitoring the toxicity of nanomaterials in practical use, as nanomaterials or nanozymes with high concentrations could pose a safety risk.
6. Designing and developing nanozymes or nanoprobes with good stability and test under clinical operating conditions remains a requirement.

As such, urine liquid biopsy‐based nano‐diagnostics are now poised to overcome the obstacles and we expect them to have seen a greater future impact on PCa precision diagnosis in the clinic. We envisage that in the next decades, advances in urine biomarker knowledge and nanotechnological breakthroughs will change nano‐diagnostics from a promising field to a powerhouse in the transformational diagnosis of PCa and other related disorders.

## 6 CONCLUSION

This review summarized the latest ongoing developments of urine PCa biomarkers merging nano‐diagnostics platforms. Early PCa detection and diagnoses have largely relied on nano‐diagnostics devices in combination with novel urinary biomarkers. PCa detections are getting easier, ultra‐sensitive, and more reliable thanks to the collaboration of materials scientists and biological engineers. We emphasized the importance of PCa biomarkers, as well as nanomaterials, in the development of sensitive nano‐diagnostics platforms. Efforts have been made to develop improved biomarkers to guide early diagnosis and slow the progression of the disease. A number of new PCa biomarkers have recently been found and brought to clinical usage, providing fresh insights into the disease for researchers and clinicians, as well as a slew of potential screening tests for PCa.

Meanwhile, great endeavors have been made in the field of nanotechnology‐based prostate cancer diagnosis, and our knowledge of the subject has vastly improved. Although only a few nano‐diagnostics‐based assays have progressed to clinical trials, a nanotechnology‐based PCa diagnosis is poised to enter the clinic in the near future with tight collaboration among scientists, engineers, and clinicians. Nanotechnology, with its high sensitivity, specificity, and multiplexed measuring capacity, has tremendous potential for improving cancer diagnosis, trailblazing the way for a better future in PCa clinical diagnosis and precision management.

## AUTHOR CONTRIBUTIONS

Literature research: Caizhi Liao, Zhihao Wu, Chan Lin, Xiaofeng Chen, Yaqun Zou, Wan Zhao, Xin Li, Guangqi Huang, Baisheng Xu, Giovanni E. Briganti, Yan Qi, Xianshu Wang, Tao Zeng, Alain Wuethrich, Hongzhi Zou. Topics discussion: Caizhi Liao, Zhihao Wu, Chan Lin, Xiaofeng Chen, Yaqun Zou, Wan Zhao, Xin Li, Guangqi Huang, Baisheng Xu, Giovanni E Briganti, Yan Qi, Xianshu Wang. Drafting of the manuscript: Caizhi Liao, Zhihao Wu, Chan Lin. Supervision and revision of the manuscript: Caizhi Liao, Tao Zeng, Alain Wuethrich, Hongzhi Zou.

## CONFLICT OF INTEREST STATEMENT

The authors declare no competing interests.
